# Health inequalities and outcomes following acute kidney injury: a systematic review & meta-analyses of observational studies

**DOI:** 10.1186/s12882-025-04391-x

**Published:** 2025-08-27

**Authors:** Christopher H. Grant, Anita Dahiya, Taylor Palechuk, Emilie Lambourg, Beatrix Tan, Ravindra L. Mehta, Neesh Pannu, Samira Bell

**Affiliations:** 1 Division of Population Health and Genomics, School of Medicine, University of Dundee, Dundee, Scotland, UK; 2 Division of Nephrology, Department of Medicine, University of Alberta, Alberta, Canada; 3 Division of Nephrology-Hypertension, Department of Medicine, University of California San Diego, California, USA

**Keywords:** Acute kidney injury, Inequalities, Inequities, Outcomes, Social determinants of health

## Abstract

**Background:**

Inequalities in health describe the uneven distribution of health outcomes that result from genetic or environmental factors. The extent to which inequalities impact on outcomes from AKI is uncertain. The aim of this systematic review and meta-analysis was to determine the impact of health inequalities on AKI outcomes.

**Methods:**

This review has been registered on PROSPERO (CRD42023422307). We included observational studies of adults who experienced at least one episode of AKI that reported outcomes stratified by sex/gender, race/ethnicity, deprivation, income, education, employment, housing, smoking, mental health conditions, geography or insurance status. The primary outcome was all-cause mortality and secondary outcomes were: progression to acute kidney disease; incident CKD; progressive CKD; AKI recovery; cardiovascular events; hospitalisations; ICU admission and hospital length of stay. The search was conducted in MEDLINE, Embase and Web of Science from inception to 10^th^ January 2024. Study selection, extraction and risk of bias (Newcastle-Ottawa) were performed independently and studies meta-analysed where possible.

**Results:**

7,312 titles/abstracts were screened, and 36 studies included (n=2,038,441). Few included data from lower-middle income countries (n=3). Evidence predominantly related to sex/gender (n=25), race/ethnicity (n=14) and deprivation (n=11). On pooling relevant studies, no sex/gender-specific differences in all-cause mortality or AKI recovery were seen. Of twelve studies reporting mortality by race/ethnicity, six found no variation by racial/ethnic group. Six of nine studies reporting mortality by socioeconomic status found deprivation was an independent predictor of death. Few studies assessed the impact of mental health (n=3), insurance (n=1), housing (n=2), geography (n=1) and smoking status (n=3) and no reports quantified the impact of income, education, employment or substance use.

**Conclusion:**

This systematic review highlights a lack of evidence related to inequalities and AKI. Further studies are required to address these gaps and achieve progress towards equitable kidney health.

**Clinical trial number:**

Not applicable.

**Supplementary Information:**

The online version contains supplementary material available at 10.1186/s12882-025-04391-x.

## Background

Inequalities in health describe the uneven distribution of health outcomes that result from genetic or environmental factors [1] which may be unavoidable or considered preventable, thereby leading to inequity [2, 3]. There is increasing recognition that health outcomes, including those related to kidney disease [4, 5] are disproportionately affected by circumstances beyond the control of the individual. These social determinants of health (SDOH) represent the socioeconomic context within which people are born, grow, work, live and age [6, 7]. They are non-medical factors (e.g. income, education, employment, housing, and discrimination) which are the result of politics, economics and public policy [8, 9]. The resulting differences in health outcomes occur between and within countries in both low and high-income settings even in spite of the presence of universal health coverage [10].

Acute Kidney Injury (AKI) is associated with high morbidity and mortality [11–15] particularly in resource poor settings [16–18]. There are regional disparities in AKI care with sub-optimal recognition, management and availability of specialist services impacting outcomes for patients [19–22].

Understanding health inequalities in the context of AKI is essential given the prognostic value of prompt recognition and management [23, 24]. Health seeking behaviours during an episode of acute illness and AKI are influenced by various factors including sex, geography and affordability of care [25, 26]. Access to appropriate diagnostics and management expertise for effective treatment is contingent upon the availability of affordable care of sufficient quality [27]. Health inequities have been described in relation to the aetiology and incidence of AKI, but the extent to which the SDOH impact on outcomes from AKI is uncertain.

The aim of this study was to systematically review the literature to determine the impact of health inequalities, including the SDOH and individual lifestyle factors, on outcomes among adults with AKI.

## Methods

This review was prospectively registered on PROSPERO (registration number: CRD42023422307) and reported in accordance with the Preferred Reporting Items for Systematic Reviews and Meta-Analyses (PRISMA) 2020 statement [28].

### Eligibility criteria

Primary observational studies of adult patients (≥16 years old) who suffered at least one episode of AKI that reported subsequent outcomes with stratification of results or subgroup analyses according to any of the following comparators of interest were eligible for inclusion: sex/gender, race/ethnicity, socioeconomic deprivation, income, education, employment, housing, smoking status, mental health conditions (i.e. dementia, depression, substance use disorders or bipolar disorder), geography (i.e. rural vs urban) or healthcare insurance status. The primary outcome of interest was all-cause mortality at any time post-AKI. Secondary outcomes were as defined by the primary reports at any time point following AKI. They included; progression to acute kidney disease (AKD), incident chronic kidney disease (CKD), progressive CKD, recovery from AKI, cardiovascular events, hospitalisations or re-hospitalisations, intensive care unit (ICU) admission and hospital length of stay. Studies were required to define AKI as an elevation of serum creatinine or initiation of acute renal replacement therapy as per consensus guidelines (i.e. KDIGO 2012, AKIN 2007 or RIFLE 2006) [23, 29, 30] or via administrative coding (i.e. ICD-10 diagnostic codes). Studies of patients with treated end stage kidney disease (ESKD), interventional or non-primary observational studies (e.g. narrative reviews) and those published in abstract form or non-English language were excluded. Full details of inclusion and exclusion criteria are provided in Supplementary Information S1.

### Search strategy & study selection

An electronic search strategy was developed in collaboration with a research librarian at the University of Alberta (JK) using a combination of free text words and MeSH terms. The search strategy included terms related to AKI, comparators of interest and observational study design. The search was developed and conducted in MEDLINE (Ovid), Embase (Ovid) and Web of Science Core Collection databases to identify all relevant articles from inception up to and including 10^th^ January 2024. The full search strategy for all databases is available in the supplementary materials (Supplementary Table S1). Six reviewers (EL, AD, CHG, TP, NP, SB) independently screened article titles and abstracts using Covidence [31] software to identify articles that potentially met eligibility criteria. Relevant articles were retrieved in full for independent review by two of three researchers (AD, CHG or TP) and studies meeting our eligibility criteria were selected for inclusion. Lastly, bibliographies of included studies were hand searched for additional citations. Discrepancies were resolved by consensus and/or discussion with a third reviewer (NP or SB).

### Data extraction

Data were extracted independently by two of four reviewers (AD, CHG, TP or BT) and exported for analysis. Data recorded included study characteristics (e.g. design, setting, sample size, missing data), measurement of exposure status (e.g. baseline serum creatinine, AKI definition, statistical analysis), patient characteristics (e.g. age, sex/gender, race/ethnicity, relevant sociodemographic factors, comorbidities, significant confounders), and outcome ascertainment (e.g. timing, effect measure, data source). Missing data were described for each study as applicable. Discrepancies in extracted data were resolved by consensus and/or discussion with a third reviewer (NP or SB).

### Risk of bias

The Newcastle-Ottawa Scale was used to assess risk of bias with relevant adaptation for design (see Supplementary Information S2) [32]. Each study was assessed by two of four independent reviewers (AD, CHG, TP or BT) with discrepancies resolved by consensus and/or discussion with a third reviewer (NP or SB).

### Data synthesis

Results were grouped according to our comparators and outcomes of interest with statistical synthesis of individual outcomes by means of meta-analysis, using study level event rates, where possible. Meta-analyses were performed using Cochrane systematic review manager, *RevMan*, software (Version 8.1.1) [33] for studies reporting mortality and AKI recovery stratified by sex/gender [33]. Random-effects meta-analysis through inverse variance was performed for all-cause mortality. If different mortality outcomes were reported in a single study, then the number of deaths by the latest time point were included in the meta-analysis (e.g. in-hospital rather than in ICU mortality). Sensitivity analyses were performed limiting the included studies to those at low risk of bias. Subgroup analyses to investigate heterogeneity were performed to assess the impact of study size and risk of bias. Heterogeneity was otherwise investigated through the I^2^ statistic. Publication bias was assessed through funnel plot analysis via R statistical software package [34]. Meta-analysis was performed for AKI recovery events reported by sex/gender using the Mantel Haenszel approach given the size of the included studies data [35]. If different recovery outcomes were reported in a single study (e.g. partial or complete recovery), ‘any recovery event’ as the result of interest was selected. Results unable to be meta-analysed due to missing event rate(s) or methodological heterogeneity were described narratively and according to direction of effect measures reported with results displayed in tabular form consistent with in text description.

## Results

### Study selection

**Fig. 1:**
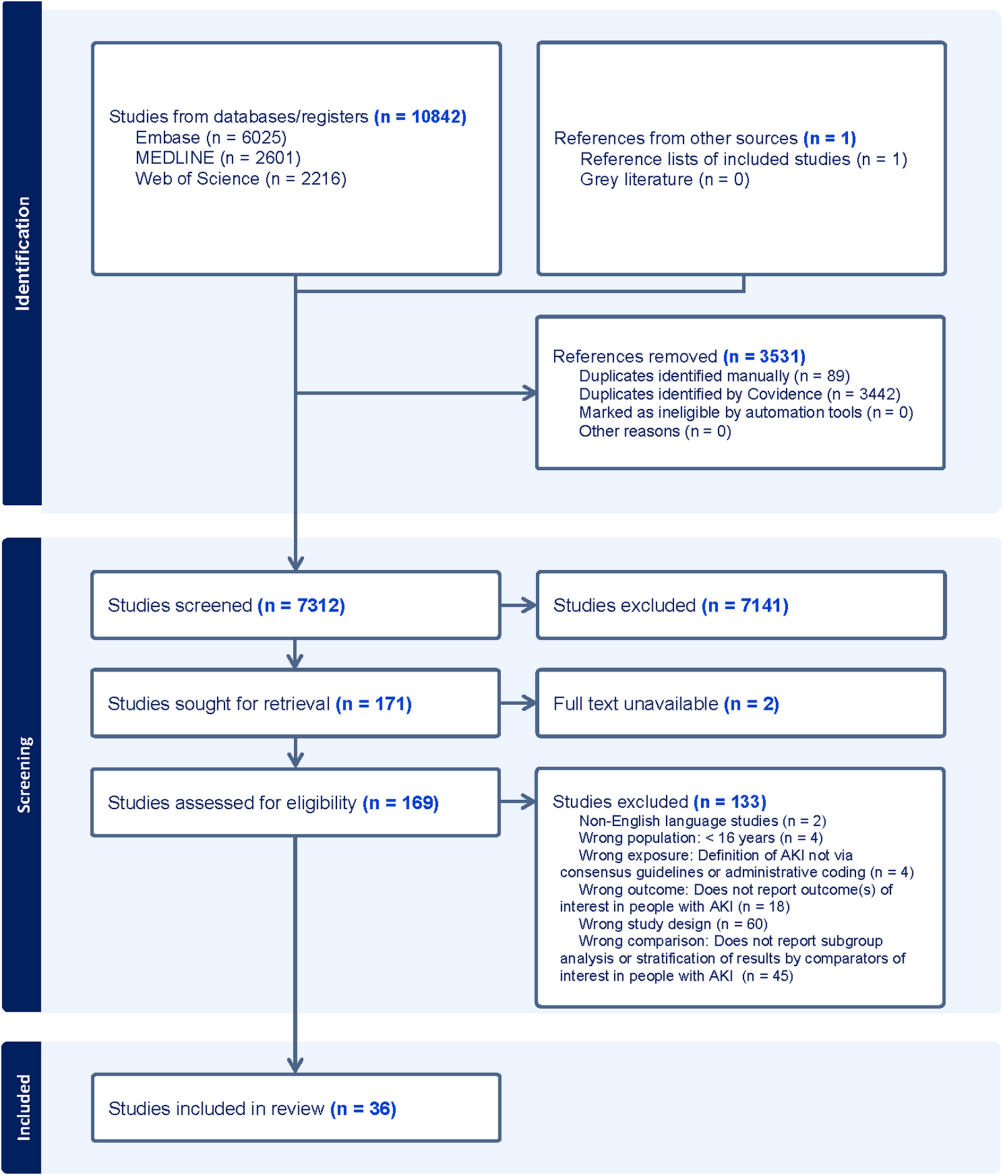
PRISMA flow diagram of studies screened, reviewed, included and excluded

Figure 1 represents the PRISMA flow diagram detailing the study selection process.

10,843 were identified by the search of which 7,312 titles/abstracts were screened for potential relevance. One hundred and sixty-nine full texts were sourced for eligibility assessment and 36 studies [35–71] were included in the review.

### Characteristics of included studies

**Fig. 2:**
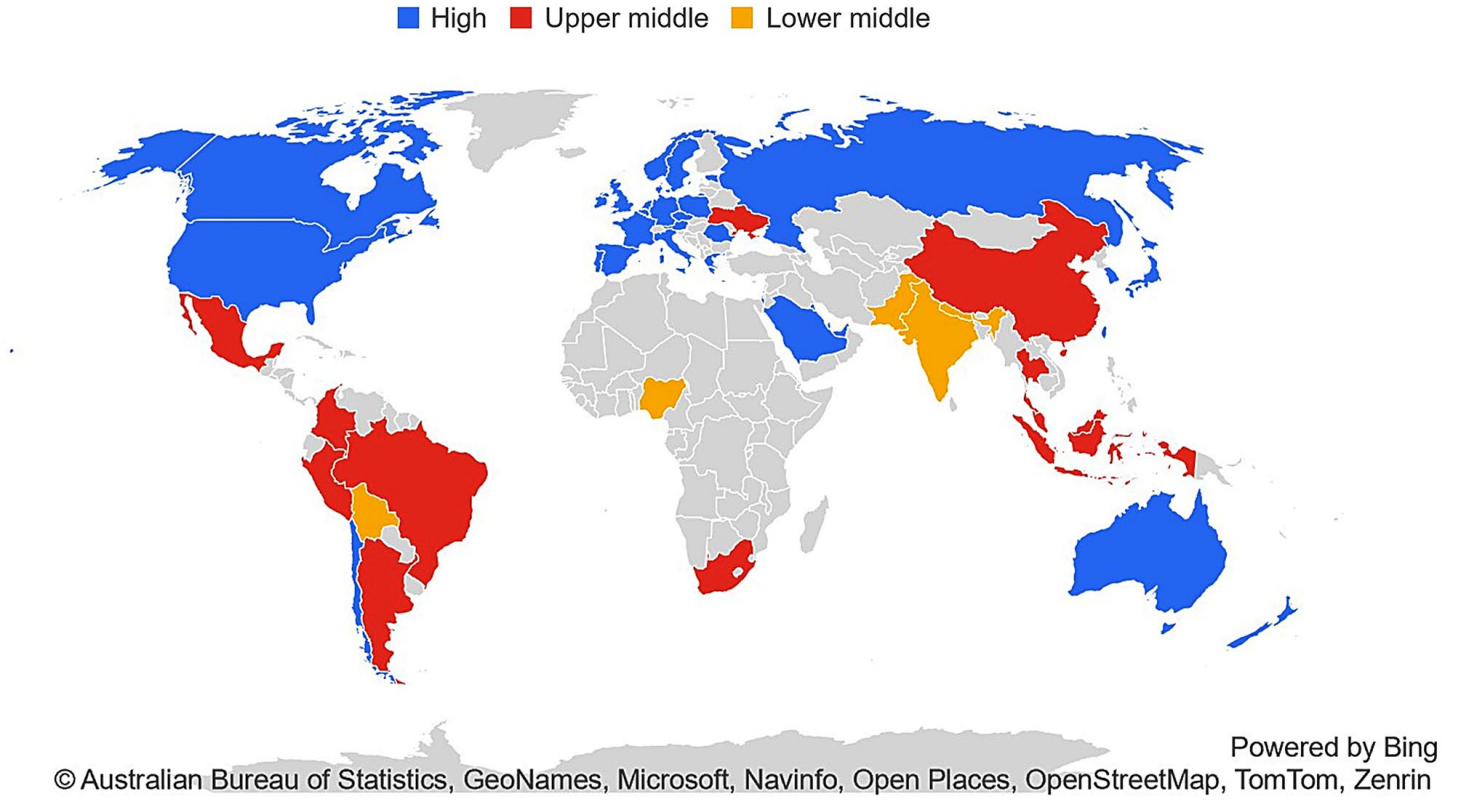
Global map of data from included studies by country income level

**Fig. 3:**
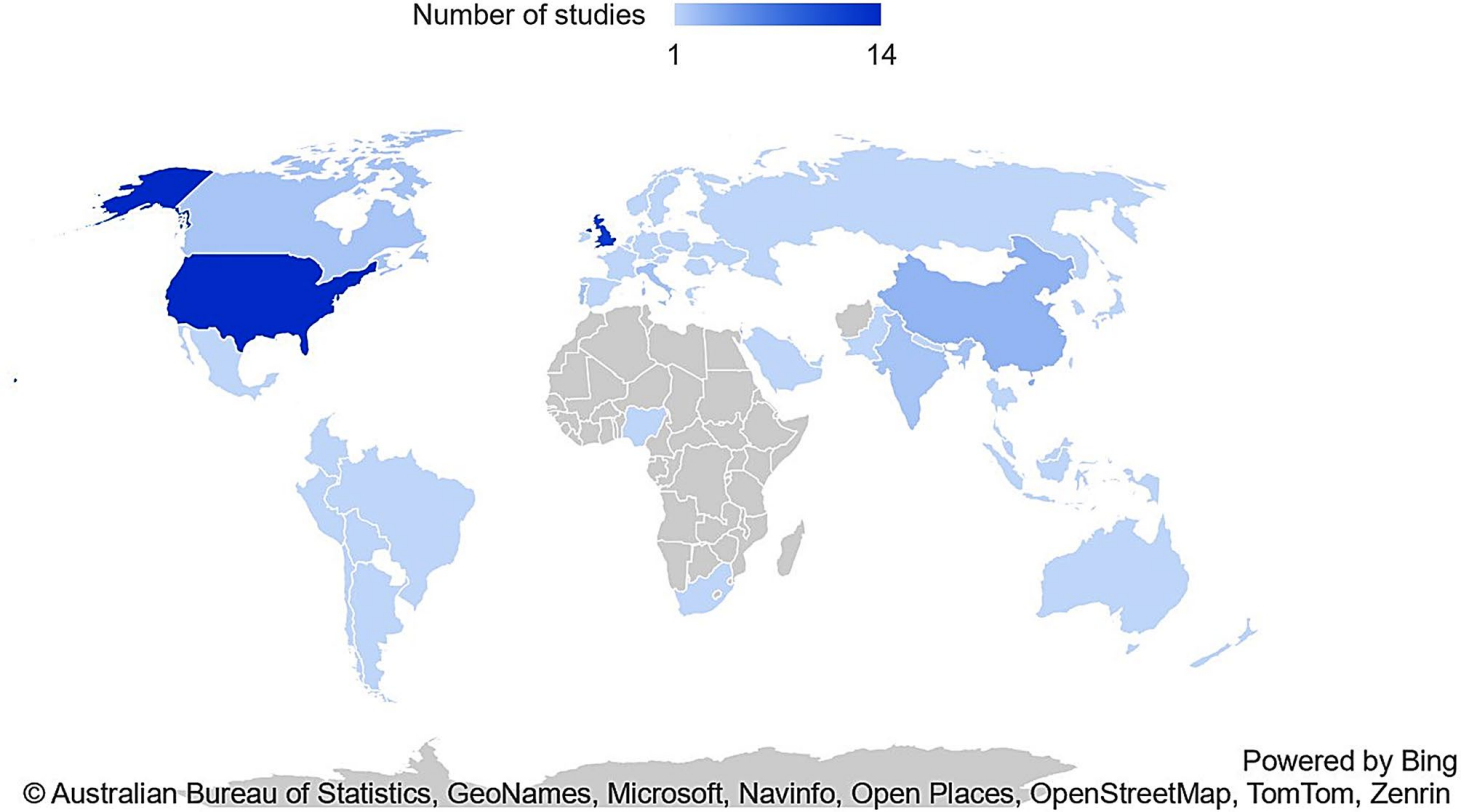
Global heat map of the number of included studies by individual country

**Fig. 4:**
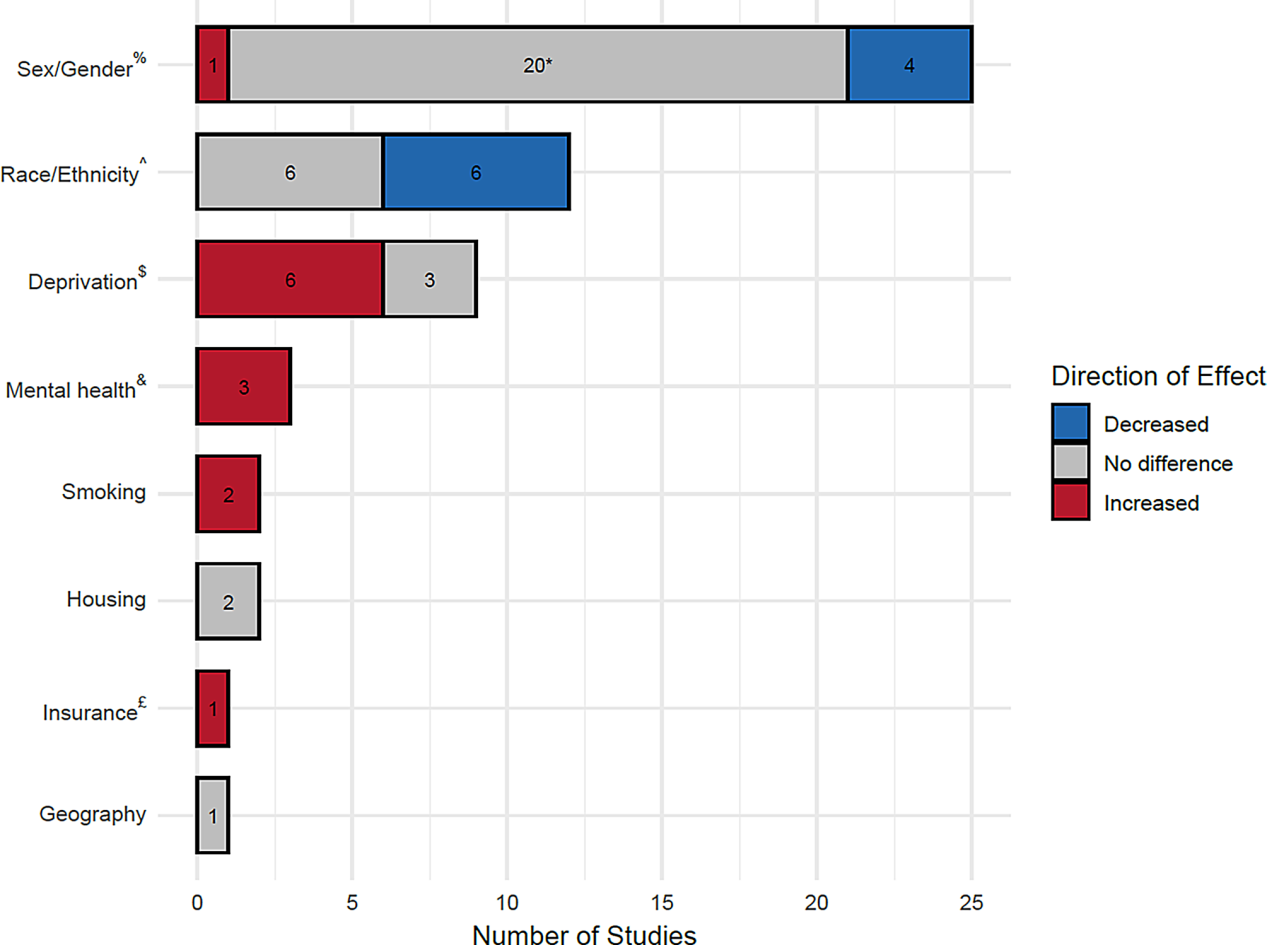
Summary results of included studies for mortality by comparators of interest. Numbers refer to the number of studies with a particular direction of effect. ^%^ Female vs male. *14 of these 20 studies were included in random-effects meta-analysis which showed no sex/gender-specific differences in mortality. ^^^ 6 studies showed reduced mortality amongst Black patients compared to other racial/ethnic groups. ^$^ Most vs least deprived. ^&^ 1 study showed increased mortality for depression vs no depression. 2 studies showed increased mortality for dementia vs no dementia. ^£^ Lack of insurance vs insurance cover

Thirty-six studies were included containing data from 2,038,441 participants with AKI. [36–71] Studies were predominantly from high income countries (HIC) (*n* = 31) [36–38, 39–47, 48–55, 57–66, 68, 70, 71]. Only three studies contained data from lower-middle income (LMIC) countries [56, 67, 69]. No studies contained data from low income countries (LIC) based on the World Bank classification [72] (see Figs. 2 and 3. Basic descriptors of all included studies are highlighted in Table 1. Summary results for the primary outcome are shown in Fig. 4. Comparators of interest reported include sex/gender (*n* = 25) (see Table 2), race/ethnicity (*n* = 14) (see Table 3), socioeconomic deprivation (*n* = 11) (see Table 4), housing (*n* = 2) [49, 62], smoking status (*n* = 3) [37, 56, 66], mental health conditions of interest (*n* = 3) [36, 42, 49], geography (*n* = 1) [47] and healthcare insurance (*n* = 1) [39] (see Table 5). No reports were identified which compared outcomes by income, education or employment.

**Table 1:** Basic descriptors of included studies, *n* = 36

Reference		Country	Study design	Study Dates	Total Sample Size	AKI definition	Setting	Health inequities explored
Balogun (2017) [36]		USA	Retrospective cohort	Jan. 2002 - Dec. 2012	11,425	KDIGO criteria	Adults admitted to the University of Virginia Medical Center (Charlottesville, VA) with AKI	Mental health conditions (depression)
Chen (2023) [37]		USA	Prospective cohort	1987–2019	14,571	Administrative coding (ICD-9-CM: 584.X or ICD-10-CM: N17.X)	Adults aged 45–64 enrolled in the Atherosclerosis Risk in Communities Study from 4 US communities (Forsyth County, NC; Jackson, MS; Washington County, MD; Minneapolis, MN)	Smoking status
Egbuche (2021) [38]		USA	Retrospective cohort	Feb. 2020 - Aug. 2020	267	KDIGO criteria	Adults seen at Grady Health System hospitals (Atlanta, GA) who were hospitalised for confirmed COVID-19	Race
Fan (2019) [39]		China	Retrospective cohort	Apr. 2013 - Mar. 2018	167	RIFLE criteria	Adults admitted to the Ningbo First Hospital (Ningbo, Zhejiang) ICU with sepsis diagnosed with AKI	Health insurance
Fisher (2020) [40]		USA	Retrospective cohort	Mar. 2020 - May 2020	4,234	KDIGO criteria	Adults within the Montefiore Health System (The Bronx, NY) who were hospitalised with confirmed COVID-19	Sex, race, ethnicity
Frydman (2022) [41]		Israel	Retrospective cohort	Jan. 2008 - Dec. 2019	2,944	KDIGO criteria	Adults admitted to the Tel-Aviv Sourasky Medical (Tel Aviv, Israel) Center cardiac ICU after STEMI diagnosis	Sex
Griffin (2023) [42]		USA	Retrospective cohort	Jan. 2013 - Dec. 2019	182,683	KDIGO criteria	Adults with an acute Veterans Affairs hospital admission with AKI across the US	Race, mental health condition (dementia)
Gupta (2021) [43]		USA	Prospective cohort	Mar. 2020 - Aug. 2020	3,099	KDIGO criteria	Adults admitted to ICUs across the US with confirmed COVID-19 at risk for AKI	Sex, race
Hassan (2021) [44]		USA	Retrospective cohort	Jan. 2001 - Dec. 2015	11,567	KDIGO criteria	Adults admitted to the University of Virginia Medical Center (Charlottesville, VA) with AKI	Race
Holmes (2019) [45]		UK	Retrospective cohort	Mar. 2015 - Jan. 2017	57,654	Electronic AKI alert that compares current and historic creatinine levels based on KDIGO criteria	Adults across Wales that triggered an electronic AKI alert	Social deprivation
Horne (2017) [46]		UK	Prospective cohort	Sep. 2011 - Oct. 2015	300	AKIN criteria	Adults admitted to the Royal Derby Hospital (Derby, UK) screened for AKI	Sex
Hounkpatin (2020) [71]		UK	Retrospective cohort	Oct. 2016 - Sept. 2018	580,940	NHS e-alert algorithm based on KDIGO criteria	Adults across Hampshire that triggered one or more AKI alert	Sex, social deprivation
Jensen (2023) [47]		Denmark	Retrospective cohort	Jan. 2017 - NR (365 after index hospitalisation)	58,356	KDIGO criteria	Adults with one or more AKI episodes seen throughout primary care and hospitals across Denmark	Geography
Kang (2020) [48]		China	Cross-sectional	Jan. 2013, Jul. 2013 (not inclusive)	258	KDIGO criteria during hospitalisation following administration of chemotherapy	Adults with CR-AKI treated in hospitals across China	Sex
Kolhe (2016) [50]		UK	Retrospective cohort	Apr. 1998 - Mar. 2013	1,136,167	Administrative coding (ICD-10: N17.0-N17.2, N17.8-N17.9)	Adults hospitalised with AKI within England’s NHS HES database	Sex, ethnicity
Kolhe (2020) [49]		UK	Retrospective cohort	Mar. 2020 - May 2020	4,535	KDIGO criteria	Adults admitted to acute hospitals serving Derbyshire and Staffordshire with suspected COVID-19 infection	Sex, ethnicity, mental health condition (dementia), housing
Liu (2019) [51]		USA	Retrospective cohort	Jan. 2006 - Dec. 2014	38,659	KDIGO criteria	Adults hospitalised at Kaiser Permanente hospitals in Northern California who survived to discharge	Sex, race, ethnicity
Lopes (2010) [52]		Portugal	Retrospective cohort	Jul. 2002 - Jun. 2007	234	RIFLE criteria	Adults admitted to the Hospital de Santa Maria (Lisbon) Infectious Disease ICU with sepsis	Sex, ethnicity
Magadi (2023) [53]		UK	Retrospective cohort	Jan. 2019 - Dec. 2019	93,196	KDIGO criteria	Adults admitted to acute NHS healthcare hospital trusts across England that triggered an AKI alert within hospital	Sex, ethnicity, social deprivation
Mathioudakis (2016) [54]		USA	Cross-sectional	2000–2010	276,138	AKIN criteria and administrative coding (ICD-9-CM: 584.0–584.9)	Adults hospitalised with AKI across the US	Race
Mitter (2010) [55]		USA	Retrospective cohort	Jan. 1995 - Dec. 2006	9,461	RIFLE criteria within the first 7 days post-op	Adults who underwent CABG and/or cardiac valve surgery at Johns Hopkins Hospital (Baltimore, MD)	Sex
Mohammed (2018) [56]		India	Retrospective cohort	Jan. 2012 - Dec. 2012	528	Administrative coding (ICD-10: N17.9)	Adults admitted to a tertiary hospital with AKI in southern India	Gender, smoking status
Peng (2022) [57]		USA	Retrospective cohort	2008–2019	6,463	KDIGO criteria within 7 days of ICU admission	Adults admitted to the Beth Israel Deaconess Medical Center (Boston, MA) ICU with sepsis	Gender
Peracha (2022) [58]		UK	Retrospective cohort	Jan. 2017 - Dec. 2018	250,504	KDIGO criteria at least 2 days following hospital admission	Adults admitted to acute healthcare hospital trusts across England with PH-AKI	Sex, ethnicity, social deprivation
Phillips (2018) [59]		UK	Retrospective cohort	Mar. 2015 - Nov. 2017	57,645	Electronic AKI alert that compares current and historic creatinine levels based on KDIGO criteria	Adults that triggered an AKI alert in hospital or community across Wales	Social deprivation
Pistolesi (2016) [60]		Italy	Retrospective cohort	1997–2012	264	RIFLE criteria at CRRT initiation	Adults who underwent CRRT for at least 48 hours for severe CS-AKI in a university hospital heart surgery ICU	Sex
Rimes-Stigare (2018) [61]		Sweden	Prospective cohort	Sep. 2008 - May 2011	201	RIFLE criteria	Adults admitted to a mixed ICU with AKI that survived to discharge	Sex
Roushani (2022) [62]		Canada	Prospective cohort	Mar. 2020 - Jan. 2021	271	KDIGO criteria (AKI-RRT only) via prospective collection	Adults with AKI-RRT registered in the ORN COVID-19 data toolkit	Sex, ethnicity, social deprivation, income, geography, housing
Sawhney (2023) [63]		UK	Retrospective cohort	2009 - Jul. 2021	142,455	KDIGO criteria	Adults with new presentations of kidney disease and AKI across Grampian (Scotland)	Sex
Shah (2020) [64]		USA	Retrospective cohort	Jan. 2005 - Dec. 2014	1,045,540	Administrative coding (tubular necrosis - no recovery CMS form 2728)	Adults who initiated dialysis for ESKD due to AKI prior to transplant or death within the USRDS dataset	Gender, race, ethnicity, social deprivation
Shiao (2020) [65]		Taiwan	Prospective cohort	Jul. 2014 - Oct. 2015	1,322	KDIGO criteria during admission	Adults with AKI-D receiving RRT in ICU that survived more than 48 hours after ICU admission	Income
Sykes (2019) [66]		UK	Prospective cohort	Nov. 2000 - Feb. 2013	2,287	KDIGO criteria	Adult outpatients from the Salford Kidney Study (Greater Manchester) referred to a secondary renal centre for CKD management	Gender, smoking status
Uduagbamen (2023) [67]		Nigeria	Retrospective cohort	Jan. 2015 - Dec. 2020	88	KDIGO criteria	Individuals aged 16 years or older admitted for cardiac and/or major vascular surgery in a large high-dependency hospital unit	Sex
Vallabhajosyula (2019) [68]	USA		Retrospective cohort	Jan. 2000 - Dec. 2014	440,257	Administrative coding (ICD-9-CM: 584, 584.5–584.9)	Adults admitted with AMI-CS across a nationally representative sample of US hospitals	Sex
Wainstein (2023) [69]		Multinational (64 countries)	Retrospective cohort	Jan. 2020 - Sep. 2022	32,120	KDIGO criteria	Adults hospitalised with suspected or confirmed COVID-19 requiring ICU admission	Income
Walker (2021) [70]		UK	Retrospective cohort	Jan. 2003 - Jan. 2019	3,524	KDIGO criteria and NHS England AKI algorithm	Adults with an index AKI in 2009 residing in the Tayside region (Scotland)	Sex, social deprivation

AKI: acute kidney injury; APACHE II: Acute Physiology and Chronic Health Evaluation II; BMI body mass index; CCI: Charlson Comorbidity Index; CHF: congestive heart failure; CI: confidence interval; CKD: chronic kidney disease; CLD: chronic liver disease; CT: computer tomography; eGFR: estimated glomerular filtration rate; EHR: electronic health record; EMR: electronic medical record; HES: Hospital Episode Statistics; HIV: human immunodeficiency virus; aHR/HR: adjusted/hazard ratio; ICD: International Classification of Diseases; ICU: intensive care unit; IQR: interquartile range; MI: myocardial infarction; NHDS: National Hospital Discharge Survey; NHS: National Health Service; aOR/OR: adjusted/odds ratio; RRT: renal replacement therapy; SBP: systolic blood pressure; USRDS: United States Renal Data System

**Table 2:** Characteristics & results of studies reporting outcomes stratified by sex/gender, *n* = 25

Reference, Country	Sample Size with AKI	Patient characteristics		Outcome Measure	Analysis	Main findings
		Measurement of Sex/Gender	Female n (%)			
Fisher (2020), USA [40]	2,099	Gender (male/female) collected using ICD-10-CM diagnostic codes.	911 (43.4%)	Time to in-hospital mortality	Cox proportional hazards models adjusted for: age, gender, HIV serostatus, AKI stage, race/ethnicity and comorbidities diabetes, hypertension, CKD, obesity)	Females were less likely to be associated with in-hospital mortality outcomes than males (HR = 0.71, 95% CI: 0.60–0.84, *p* < 0.001)
Frydman (2022), Israel [41]	255	Sex (male/female) was collected from patient medical records.	69 (27.1%)	Any (early + partial) recovery from AKI^$^, cardiovascular events including stent thrombosis, VT/VF and need for IAMP/inotropes, and in-hospital mortality^$^	Chi-squared tests	No significant differences were found in any recovery outcomes (71.5% vs. 62.3%, *p* = 0.16), stent thrombosis (7.0% vs. 4.3%, *p* = 0.44) or VT/VF events (18.8% vs. 10.1%, *p* = 0.10), need for IAB/inotropes (21.0% vs. 21.7%, *p* = 0.89), or in-hospital mortality (15.0% vs. 19.7%, *p* = 0.38) between males and females.
Gupta (2021), USA [43]	637	Sex (male/female) was collected via chart review	180 (28.3%)	28-day all-cause mortality since ICU admission or initiation of RRT	Multivariable logistic regression models adjusted for: age, sex, race, BMI, comorbidities (diabetes, hypertension, active malignancy, CAD, CHF, CKD), days to ICU admission, severity of illness covariates and hospital characteristics.	Males did not have significantly different outcomes than females for 28-day mortality since ICU admission (OR = 1.29, 95% CI: 0.83–2.00) or initiation of RRT (OR = 1.31, 95% CI: 0.84–2.04).
Horne (2017), UK [46]	150	Gender (male/female) was collected via hospital patient administration system	62 (41.3%)	Progressive CKD at 3 years following enrollment defined as a decrease in eGFR (≥25%) and stage	Univariate, multivariate and binary logistic regression corrected for: age, gender, diabetes, baseline and change in eGFR.	Male gender was associated with CKD progression at 3 years (OR = 2.57, 95% CI: 1.15–5.76) and remained significant after adjustments (aOR = 2.88, 95% CI: 1.06–7.79).
Hounkpatin (2020) [71]	10,028	Sex (male/female) was collected from the Care & Health Information Analytics linked primary care database	5,413 (54.0%)	All-cause mortality over a median (IQR) 234 day (119–356) follow-up following first AKI event	Cox proportional hazard models adjusted for: age, sex, comorbidities and medications.	Females were associated with a lower risk of mortality than males post first AKI (HR = 0.81, 95% CI: 0.75–0.87).
Kang (2020), China [48]	258	Sex (male/female) was collected per a nationwide AKI survey using hospital medical records of patients.	104 (40.3%)	Failure to recover from AKI^$^ and in-hospital all-cause mortality^$^	Univariate and multivariate logistic regression adjusted for: sex, age, peak AKI stage, CVD, prescription drug use, recovery, pre-existing CKD and malignant solid tumours	Male sex was not associated with failure to recover from AKI (OR = 1.67, 95% CI: 0.96–2.91, *p* = 0.07; aOR = 1.76, 95% CI: 0.99–3.13, *p* = 0.06) in recovery from AKI. Males were associated with in-hospital mortality (OR = 2.45, 95% CI: 1.11–5.42, *p* < 0.05) but was no longer significant after adjusting for covariates (aOR = 2.21, 95% CI: 0.96–5.09, *p* = 0.06).
Kolhe (2016), UK [50]	1,136,167	Gender (male/female) was extracted from coded population censuses and surveys (OPCS-4 Classification of Interventions and Procedures version 4).	523,820 (46.1%)	In-hospital all-cause mortality	Univariate and multivariate logistic regression adjusted for age, gender, ethnicity, CCI score, admission method, 5-year study period, diagnostic codes	Females were more likely to die in-hospital after AKI than males (OR = 1.11, 95% CI: 1.10–1.12, *p* < 0.05; aOR = 1.06, 95% CI: 1.05–1.07, *p* < 0.05).
Kolhe (2020), UK [49]	724	Gender (male/female) was collected from hospital electronic patient records.	331 (45.7%)	In-hospital all cause mortality^$^ and survival.	Univariate and multivariable logistic regression adjusted for: age, gender, care home residence, comorbidities, COVID-19 status, AKI characteristics and treatments	No differences in all-cause mortality outcomes were found between males and females (OR = 1.27, 95% CI: 0.88–182, *p* = 0.20). However, male gender was independently associated with survival than female (59.7% vs. 50.5%, *p* = 0.02).
Liu (2019), USA [51]	38,659	Sex (male/female) was collected from EHRs	19,137 (29.5%)	Re-hospitalization for recurrent AKI over a median 1.8 years	Cox proportional hazard models and standardised differences adjusted for: age, gender, race, ethnicity, severity of AKI, CV history, procedure history, medical history, BMI, SBP, lab findings, index hospitalisation	Male sex was associated with rehospitalization for recurrent AKI (HR = 1.10, 95% CI: 1.06–1.15).
Lopes (2010), Portugal [52]	138	Sex (male/female) was collected from unit databases and medical charts	38 (27.5%)	In-hospital survival^$^	Cox regression method adjusted for: age, gender, race, comorbidities, AKI, eGFR, APACHE II score	No differences were found with in-hospital survival outcomes between males and females (68.8% vs. 77.0%, *p* = 0.38).
Magadi (2023), UK [53]	93,196	Sex (male/female) was collected from linked NHS HES data.	48,127 (51.6%)	30-day all cause mortality^$^ from discharge	Logistic regression model adjusted for: age, sex, ethnicity, deprivation, comorbidity score, peak AKI stage, elective or emergency admission and season.	Females were less likely to die within 30-days after AKI than males (OR = 0.76, 95% CI: 0.73–0.78).
Mitter (2010), USA [55]	395	Sex (male/female) was collected by abstractors from EMRs.	160 (40.5%)	Cardiovascular events including stroke, perioperative MI and 30-day mortality^$^	Univariate logistic regression model and descriptive statistics	Females experienced postoperative stroke more than males (14.4% vs. 10.2%) but had similar counts of perioperative MI (0.0% vs. 0.8%). No differences were found in 30-day mortality outcomes in females (OR = 3.96, 95% CI: 1.86–8.44) and males (OR = 4.06, 95% CI: 2.19–7.48).
Mohammed (2018), India [56]	528	Sex (male/female) was collected from case report forms	144 (27.3%)	Incident CKD, recovery from AKI^$^ and (presume in-hospital) mortality^$^	Multivariate logistic regression and descriptive statistics adjusted for: gender, diabetes, benign prostatic hypertrophy & urinary tract infection	Mortality was increased in females than males (22.9% vs. 12.8%). No differences in recovery from AKI were found between sexes. Males had an 8-fold increase in incident CKD diagnosis (OR = 4.37, 95% CI: 1.01–18.85, *p* < 0.05; aOR = 8.35, 95% CI: 1.08–64.73, *p* < 0.05).
Peng (2022), USA [57]	5,368	Gender (man/woman) was collected from the MIMIC-IV database	2,290 (42.7%)	In-hospital^$^ and ICU mortality	Propensity score matching	No difference in ICU (19.7% vs. 20.4%, *p* = 0.60) nor in-hospital (25.6% vs. 25.8%, *p* = 0.99) mortality between men and women.
Peracha (2022), England [58]	250,504	Sex (male/female) was collected using a Master Patient Index	126,254 (50.4%)	All-cause in-hospital or 30-day mortality from discharge	Multivariable logistic regression models adjusted for: sex, age, ethnicity, deprivation, admission method, AKI alert warning level, comorbidity score, month of alert, diagnosis group and specialised nephrology services	Males were more likely to die in-hospital or within 30 days than females (OR = 1.27, 95% CI: 1.25–1.30).
Pistolesi (2016), Italy [60]	264	Gender (male/female) was collected using demographic data	72 (27.3%)	Recovery from AKI defined as independence from RRT^$^ and survival to hospital discharge^$^	Cox proportional hazard models	No differences in recovery from AKI were found between males and females (HR = 1.43, 95% CI: 0.90–2.27). Females were more likely to survive to discharge than males (HR = 1.82, 95% CI: 1.08–3.06, *p* = 0.02).
Rimes-Stigare (2018), Sweden [61]	201	Sex (male/female) was collected from ICU data system	114 (41.6%)*	Incident CKD at follow-up between 2 and 7 months who survived to 90 days	Multivariate logistic regression adjusted for: age, sex, maximum RIFLE stage and creatinine:cystatin C ratio	Females had a threefold excess in de novo CKD Model 1 (ref = male): female OR 3.0 (1.5–6.1) P 0.002/Model 2 (ref male): female OR 3.4 (1.7–6.9) P 0.001
Roushani (2022), Canada [62]	271	Sex (male/female) was collected from the Registered Persons Database	65 (24.0%)	90-day all cause mortality from RRT initiation^$^	Multivariable logistic regression models adjusted for: sex, age, geographic location, ethnicity, diabetes, type of residence, income quintile, social deprivation, baseline serum creatinine and time of initiation of RRT relative to COVID-19 diagnosis	No significant differences in likelihood of 90-day mortality were found between male and female patients (OR = 1.13, 95% CI: 0.59–2.14).
Sawhney (2023), UK [63]	41,313	Sex (male/female) was collected from linked administrative databases	1,724 (53.7%)	Incident CKD defined as eGFR < 15 mL/min/1.73 m2 for 90 days or onset of kidney replacement therapies, mortality at 1 year^$^ & 3-years and long-term mortality	Univariate and multivariate logistic regression models adjusted for age, sex and comorbidities (cancer, CHD, HF, stroke, AFib, PAD, diabetes, hypertension, liver disease, CPD)	Males (HR = 1.25, 95% CI: 1.17–1.34) were associated with a greater risk of long-term mortality than females (HR = 1.12, 95% CI: 1.04–1.21; P interaction = 0.03). No significant differences were found between sexes on progression of CKD (male: HR = 1.24, 95% CI: 0.99–1.55; female: HR = 1.00, 95% CI: 0.78–1.29; P interaction = 0.21).
Shah (2020), USA [64]	32,598	Gender (man/woman) was collected from USRDS patient files	13,691 (42.0%)	Recovery from AKI defined as discontinuation of dialysis due to recovered kidney function within 12 months of kidney failure^$^	Fine and Gray cumulative incidence models with death as a competing risk and Cox proportional hazard models adjusted for: age, year of dialysis initiation, sex, race/ethnicity, body mass index, neighbourhood poverty, region, comorbidities, laboratory values, nursing home and nephrology care	Women were found to be associated with lower rates of recovery from AKI within 12 months than men (aHR = 0.86, 95% CI: 0.83–0.90, *p* < 0.05).
Shiao (2020), Taiwan [65]	1,322	Sex (male/female) was collected from a patient registry	479 (36.2%)	Recovery from AKI^$^	Fine and Gray regression models and descriptive statistics	Counts of those who recovered from AKI were similar between males and females (23.3% vs. 23.0%).
Sykes (2019), UK [66]	643	Sex (male/female) was self-reported via structured patient questionnaires delivered by research nurses	247 (38.4%)	Progressive CKD defined as the initiation of chronic RRT and all-cause mortality after an index or subsequent AKI events^$^	Competing risk models adjusted for: age, hospital, gender, smoking status, alcohol intake, diabetes, cardiovascular comorbidities and primary renal disease	No differences between males and females were found in progressive CKD outcomes before a second (HR = 1.27, 95% CI: 0.95–1.70, *p* = 0.10) or third AKI event (HR = 1.02, 95% CI: 0.44–2.39, *p* = 0.96). Similarly, female sex was not a significant risk factor for mortality following a first (HR = 1.02, 95% CI: 0.67–1.57, *p* = 0.91) or second AKI event (HR = 0.53, 95% CI: 0.25–1.10, *p* = 0.09).
Uduagbamen (2023), Nigeria [67]	88	Sex (male/female) was collected from perioperative charts, case notes, ICU charts and hospital database	22 (25.0%)	Recovery from AKI as defined as non-recovery of kidney function^$^ and severity of AKI grade and hospital length of stay. All-cause mortality up to post-op day 30^$^	Multiple logistic regression adjusted for: sex, age, preoperative kidney dysfunction, AKI stage III, dialysis	No significant differences in recovery from AKI were found between males and females (aOR = 3.12, 95% CI: 0.56–8.22, *p* = 0.04^£^). Similarly, hospital length of stay of less than 7 days was not significantly different between sexes (OR = 2.20, 95% CI: 0.61–3.38, *p* = 0.05^£^). No sex-specific differences in all-cause mortality were reported (unadjusted OR 5.9 95% CI 0.89‑20.71 P 0.02^£^)
Vallabhajosyula (2019), USA [68]	155,610	Sex (male/female) was collected from an administrative database	56,486 (36.3%)	All-cause in-hospital mortality^$^ and hospital length of stay	Chi-squared tests and multivariable logistic regression adjusted for: age, comorbidities, CKD, race, socioeconomic status, hospital characteristics, acute organ failure, cardiac arrest, cardiac and non-cardiac interventions	Females were more likely to die in hospital than males (OR = 1.28, 95% CI: 1.25–1.31, *p* < 0.001) and remained significant after adjusting for covariates (aOR = 1.16, 95% CI: 1.14–1.19, *p* < 0.001). Females were reported to have a shorter length of stay than men (12 ± 14 days vs. 13 ± 14 days, *p* < 0.001) in unadjusted analyses.
Walker (2021), UK [70]	3,524	Adults with AKI in the Tayside region	2,003 (56.8%)	All-cause mortality over a median 3.17 year follow-up from the date of index AKI	Cox proportional hazard models adjusted for: age, sex, diabetes, AKI stage, social deprivation (SIMD), previous MI, PVD, cerebrovascular disease, prescription drug use in 3 months leading up to AKI	Males were found to have an increased risk of mortality than females (aHR = 1.22, 95% CI: 1.12–1.32).

AFib: atrial fibrillation; AKI: acute kidney injury; AKI-RRT: acute kidney injury requiring renal replacement therapy; APACHE II: Acute Physiology and Chronic Health Evaluation II; BMI body mass index; CAD: coronary artery disease; CCI: Charlson Comorbidity Index; CHD: congenital heart disease; CHF: congestive heart failure; CI: confidence interval; CKD: chronic kidney disease; CPD: cardiopulmonary disease; CVD: cardiovascular disease; eGFR: estimated glomerular filtration rate; EHR: electronic health record; EMR: electronic medical record; ESKD: end-stage kidney disease; HES: Hospital Episode Statistics; HF: heart failure; HIV: human immunodeficiency virus; aHR/HR: adjusted/hazard ratio; IABP: intra-aortic balloon pump; ICU: intensive care unit; IQR: interquartile range; MI: myocardial infarction; NHS: National Health Service; aOR/OR: adjusted/odds ratio; PAD: pulmonary artery disease; PVD: peripheral vascular disease; RRT: renal replacement therapy; SBP: systolic blood pressure; SIMD: Scottish Index of Multiple Deprivation; STEMI: ST elevation myocardial infarction; USRDS: United States Renal Data System; VF: ventricular fibrillation; VT: ventricular tachycardia

* Baseline characteristics of all recruited patients with AKI, *n* = 274

$ Result (event rate) included in meta-analysis of either mortality or AKI recovery as relevant

£ As reported in the primary paper despite not appearing to be statistically correct. Authors were contacted for clarification

**Table 3:** Characteristics & results of studies reporting outcomes stratified by race/ethnicity, *n* = 14

Reference, Country	Sample Size with AKI	Patient Characteristics		Outcome Measure	Analysis	Main findings
		Measurement of Race/Ethnicity	Race/Ethnicity Categories (n/%)			
Egbuche (2021), USA [38]	75	Race and ethnicity was stratified into 2 subgroups: Black and non-Black (Hispanic, Asian, white, other) by self-report via EMR.	Black: 66 (88.0%) Non-Black: 9 (12.0%)	Cardiovascular events defined as a primary composite outcome of cardiac events plus death, MI event, ICU admission, in-hospital mortality	Univariate analysis (Breslow-Day), binary logistic regression and multivariable analysis adjusted for: age, gender and comorbidities	No significant differences were found between Black and non-Black patients in primary composite cardiovascular events (aOR = 0.30 95% CI: 0.04–1.86), MI (aOR = 0.66, 95% CI: 0.14–3.12), ICU admission (aOR = 0.80, 95% CI: 0.20–3.25) or in-hospital mortality outcomes (aOR = 1.40, 95% CI: 0.26–7.50).
Fisher (2021), USA [40]	2,099	Ethnicity was collected using ICD-10-CM diagnostic codes.	Black, non-Hispanic: 828 (39.5%) White, non-Hispanic: 175 (8.3%) Hispanic (any race): 695 (33.1%) Asian and Pacific Islander: 59 (2.8%) Other: 157 (7.5%) Unknown: 185 (8.8%)	Time to in-hospital mortality	Cox proportional hazards models adjusted for: age, gender, HIV serostatus, AKI stage, race/ethnicity and comorbidities (diabetes, hypertension, CKD, obesity)	Black, non-Hispanic patients were associated with a decreased risk of in-hospital mortality than white, non-Hispanic patients (aHR = 0.71, 95% CI: 0.54–0.95, *p* = 0.02) while there were no significant differences found between Hispanic and white, non-Hispanic patients (aHR = 0.79, 95% CI: 0.59–1.06).
Griffin (2023), USA [42]	182,683	Race was stratified into 2 subgroups: Black and non-Black using inpatient files via EHR.	Black: 40466 (22.2%) Non-Black: 142,217 (77.8%)	1-year mortality	Multivariable analysis using bootstrap resampling to find an appropriate non-parsimonious model adjusting for 30 variables including: Black race, comorbidities, inpatient lab variables, AKI stage.	Black patients were found to be less likely to die within 1 year of AKI than non-Black patients (aOR = 0.92, 95% CI: 0.87–0.97, *p* < 0.001).
Gupta (2021), USA [43]	637	Race and ethnicity was collected via chart review and stratified into white and non-white groups.	White: 173 (27.2%) Black: 280 (44.0%). Asian: 27 (4.2%) Other/unknown: 157 (24.6%)	28-day all-cause mortality since ICU admission or initiation of RRT	Multivariable logistic regression models adjusted for: age, sex, race, BMI, comorbidities, days to ICU admission, severity of illness covariates and hospital characteristics.	No differences in mortality outcomes were found between white and non-white (Black, Asian, other/unknown, Hispanic) patients within 28-days of ICU admission (OR = 1.05, 95% CI: 0.68–1.62) nor initiation of RRT (OR = 1.08, 95% CI: 0.69–1.69).
Hassan(2021), USA [44]	11,567	Race was collected via a clinical data repository	Black: 2,116 (18.3%) White: 9,451 (81.7%)	Hospital length of stay, in-hospital mortality, 90-day mortality*, long-term survival	Logistic regression analysis, univariate analysis, Kaplan-Meier survival model and Cox proportional hazards adjusted for: age, gender, Deyo-Charlson index, comorbid conditions (cancer, CKD, diabetes, gastrointestinal bleed, pneumonia, CHF, cardiovascular events), mechanical ventilator use	Compared to white patients, Black patients had significantly shorter median lengths of hospital stays than white patients (10 days, IQR: 6, 17 vs. 11 days, IQR: 7, 20 *p* < 0.001). Black patients were significantly less likely to die in-hospital (OR = 0.82, 95% CI: 0.70–0.96, *p* = 0.015) or within 90 days (OR = 0.64, 95% CI: 0.46–0.89, *p* = 0.008). Black patients also had lower long-term mortality (HR = 0.87, 95% CI: 0.77–0.99, *p* = 0.030) and had greater median survival than white patients (39.7 vs. 24.8 months, *p* < 0.001).
Kolhe (2016), UK [50]	1,136,167	Ethnicity was extracted from coded population censuses and surveys (OPCS-4)	White: 944,074 (83.1%) Mixed: 2,915 (0.3%) Asian: 32870 (2.9%) Black: 20347 (1.8%) Other: 11584 (1.0%) Unknown: 124,377 (10.9%)	In-hospital mortality	Univariate and multivariate logistic regression adjusted for age, gender, ethnicity, CCI score, admission method, 5-year study period, diagnostic codes	Compared to white patients, patients with mixed (aOR = 0.74, 95% CI: 0.67–0.82, *p* < 0.05), Asian (aOR = 0.73, 95% CI: 0.71–0.75, *p* < 0.05), Black (aOR = 0.60, 95% CI: 0.58–0.62, *p* < 0.05) or other ethnicities (aOR = 0.85, 95% CI: 0.82–0.89, *p* < 0.05) were associated with lower likelihood of in-hospital mortality. Conversely, patients with unknown ethnicity were associated with greater likelihood of in-hospital mortality (aOR = 1.35, 95% CI: 1.34–1.37, *p* < 0.05).
Kolhe (2020), UK [49]	724	Race was collected from in-hospital self-reports.	White: 592 (81.8%), Asian: 31 (4.3%), Black: 14 (1.9%), Not reported: 75 (10.3%) Missing: 12 (1.7%)	All cause mortality and survival.	Univariate and multivariable logistic regression adjusted for: age, gender, care home residence, comorbidities, COVID-19 status, AKI characteristics and treatments	No significant differences in ethnicity were found to be risk factors of mortality in patients with AKI (White: reference; Asian: OR = 1.49, 95% CI: 0.62–3.58; Black: OR = 2.77, 95% CI: 0.75–10.24; not reported: OR = 1.52, 95% CI: 0.85–2.73). Ethnicity was not found to be a significant risk factor between AKI survivors and non-survivors (*p* = 0.153).
Liu (2019), USA [51]	38,659	Race and ethnicity was collected from self-reported data via EHR.	White: 27669 (71.6%) Black/African American: 4,404 (11.4%) Asian and Pacific Islander: 4,675 (12.1%) Native American: 265 (0.7%) Unknown: 1,646 (4.3%) Hispanic ethnicity: 5,538 (14.3%)	Re-hospitalization for recurrent AKI over a median 1.8 years	Cox proportional hazard models and standardised differences adjusted for: age, gender, race, ethnicity, severity of AKI, cardiovascular history, procedure history, medical history, BMI, SBP, lab findings, index hospitalisation	Black/African American and Hispanic patients were both found to have a modest increase in risk of re-hospitalization due to recurrent AKI than white patients, respectively (aHR = 1.15, 95% CI: 1.08–1.22, *p* < 0.05; aHR = 1.11, 95% CI: 1.05–1.18, *p* < 0.05). Patients with unknown ethnicity were found to have a reduced risk of re-hospitalization (aHR = 0.76, 95% CI: 0.67–0.86). No other racial group was found to be significantly different from white patients.
Lopes (2010), Portugal [52]	138	Ethnicity was reported as the percentage of Caucasian patients from patient chart databases	Caucasian: 118 (85.5%) Non-Caucasian: 20 (14.5%)	All-cause mortality	Cox regression method adjusted for: age, gender, race, comorbidities, AKI, eGFR, APACHE II score	There were no differences between AKI non-survivors and survivors in-hospital among Caucasian patients (87.0% vs. 83.6%, *p* = 0.748).
Magadi (2023), UK [53]	93,196	Ethnicity was collected from linked NHS HES data.	White: 77853 (91.4%) Asian: 4,042 (4.7%) Black: 1,772 (2.1%) Mixed: 362 (0.4%) Other: 1,178 (1.4%)	30-day all cause mortality from discharge	Logistic regression model adjusted for: age, sex, ethnicity, deprivation, comorbidity score, peak AKI stage, elective or emergency admission and season.	Compared to white patients, Asian (OR = 0.81, 95% CI: 0.74–0.88, *p* < 0.05), Black (OR = 0.70, 95% CI: 0.61–0.80, *p* < 0.05) and patients with other ethnicities (OR = 0.82, 95% CI: 0.70–0.96, *p* < 0.05) were associated with lower odds of 30-day mortality. Patients recorded with missing ethnicity had greater odds of 30-day mortality (OR = 1.12, 95% CI: 1.06–1.19, *p* < 0.05) while mixed patients were not significantly different than white patients.
Mathioudakis (2016), USA [54]	13,748	Race was collected from NHDS restricted databases	Black: 4,368 (31.8%); White: 9,380 (68.2%)	Hospital length of stay, in-hospital all cause mortality	Multivariable logistic regression and multivariable linear regression adjusted for: age, sex, and AKI-related clinical risk factors (CKD, sepsis, hypertension, hypotension, length of stay, acute MI, CHF, angiography, CT scan, and CLD), sociodemographic factors, admission source, payer source, race/payer interaction, hospital region and size	No significant differences in length of hospital stay were found between Black and white patients (5 days, IQR: 3, 8 vs. 5 days, IQR: 3, 7; *p*-0.71). Similarly, in-hospital mortality rates were similar regardless of race (OR = 0.35, 95% CI: 0.04–3.47).
Peracha (2022), UK [58]	250,504	Ethnicity was collected using a Master Patient Index	White: 226,799 (90.7%) South Asian: 8,252 (3.3%) Black: 40001 (1.6%) Other: 3,751 (1.5%) Unknown: 2,000 (0.8%) Missing: 5,001 (2.0%)	All cause in-hospital and 30-day mortality from discharge	Multivariable logistic regression models adjusted for: sex, age, ethnicity, deprivation, admission method, AKI alert warning level, comorbidity score, month of alert, diagnosis group and specialised nephrology services	Compared to white patients, South Asian (aOR = 0.78, 95% CI: 0.74–0.83), Black (aOR = 0.76, 95% CI: 0.70–0.83) and patients with unknown ethnicity (aOR = 0.84, 95% CI: 0.77–0.92) were found to have lower likelihood of in-hospital and 30-day mortality. Patients with missing (aOR = 1.58, 95% CI: 1.42–1.75) or other (aOR = 1.32, 95% CI: 1.23–1.41) ethnicity were more likely to die in-hospital or within 30-days compared to white patients.
Roushani (2022), Canada [62]	271	Race and ethnicity was stratified into 3 subgroups: white, non-white and unknown/missing through the Ontario Renal Reporting System	White: 111 (41.0%) Non-white: 99 (36.5%) Unknown/missing: 61 (22.5%)	90-day all cause mortality from RRT initiation	Multivariable logistic regression models adjusted for: sex, age, geographic location, ethnicity, diabetes, type of residence, income quintile, deprivation, baseline serum creatinine and time of initiation of RRT relative to COVID-19 diagnosis	No significant differences were found in non-white (OR = 1.13, 95% CI: 0.60–2.14) or unknown/missing ethnicity (OR = 1.15, 95% CI: 0.55–2.42) patients compared to white patients in the likelihood of mortality within 90 days of RRT initiation.
Shah (2020), USA [64]	32,598	Race and ethnicity were collected from USRDS patient files	Asian: 652 (2.0%) Black: 4,890 (15.0%) Native American: 163 (0.5%) White: 24774 (76.0%) Hispanic ethnicity: 2,282 (7.0%) Missing: 2,119 (6.5%)	Recovery from AKI defined as discontinuation of dialysis due to recovered kidney function within 12 months of kidney failure	Fine and Gray cumulative incidence models with death as a competing risk and Cox proportional hazard models adjusted for: age, year of dialysis initiation, sex, race/ethnicity, body mass index, neighbourhood poverty, region, comorbidities, laboratory values, nursing home and nephrology care	Patients from Black (aHR = 0.68, 95% CI: 0.64–0.72), Asian (aHR = 0.82, 95% CI: 0.69–0.96), Hispanic (aHR = 0.82, 95% CI: 0.76–0.89) and Native American (aHR = 0.72, 95% CI: 0.54–0.95) racial and ethnic groups were significantly less likely to recover from AKI within 12 months compared to white patients.

AKI: acute kidney injury; APACHE II: Acute Physiology and Chronic Health Evaluation II; BMI body mass index; CCI: Charlson Comorbidity Index; CHF: congestive heart failure; CI: confidence interval; CKD: chronic kidney disease; CLD: chronic liver disease; CT: computer tomography; eGFR: estimated glomerular filtration rate; EHR: electronic health record; EMR: electronic medical record; HES: Hospital Episode Statistics; HIV: human immunodeficiency virus; aHR/HR: adjusted/hazard ratio; ICD: International Classification of Diseases; ICU: intensive care unit; IQR: interquartile range; MI: myocardial infarction; NHDS: National Hospital Discharge Survey; NHS: National Health Service; aOR/OR: adjusted/odds ratio; RRT: renal replacement therapy; SBP: systolic blood pressure; USRDS: United States Renal Data System

**Table 4:** Detailed characteristics of studies reporting social deprivation, *n* = 11

Reference, Country	Sample Size with AKI	Patient Characteristics		Outcome Measure	Analysis	Main findings
		Definition of Social Deprivation	Social Deprivation Categories n (%)			
Holmes (2019), Wales [45]	57,654	WIMD score that divides Wales into geographical units grouped by postal code and weighs various social determinants of health. (1 = most deprived, 100 = least deprived)	Total: 20411 reported (35.4%) 1–25: 9,386 (46.0%) 26–50: 765 (3.7%) 51–75: 4,595 (22.5%) 76–100: 5,665 (27.8%)	90-day mortality	Multivariate Cox proportional hazard models adjusting for: age, generating a Beta correction factor to adjust each WIMD percentile population to age 60.	90-day mortality was marginally lower in the most affluent population (HR = 0.999; 95% CI: 0.998–0.999; *p* < 0.001)
Hounkpatin (2020) [71]	10,028	Socioeconomic status was defined using the 2015 IMD quintiles (1 = most deprived, 5 = least deprived)	Quintile 1 (most deprived): 1,498 (15.1%) 2: 2,006 (20.2%) 3: 1,707 (17.2%) 4: 2,077 (20.9%) 5 (least deprived): 2,658 (26.7%)	Recovery defined by comparing the lowest creatinine value within 90 and 180 days to the baseline creatinine at the time of the alert All-cause mortality assessed over a median (IQR) of 234 days (119–356) post first AKI	Logistic regression model for AKI recovery. Cox proportional hazard for all-cause mortality. Models adjusted for age, sex, comorbidity, and prescribed medications	No significant differences were found among the IMD quintiles for recovery outcomes. Compared to the least deprived patients, the risk of all-cause mortality was increased in the most deprived populations (quintiles 1: aHR = 1.20, 95% CI: 1.07–1.36; 2: aHR = 1.17, 95% CI: 1.05–1.30; 3: aHR = 1.14, 95% CI: 1.03–1.27; 4: aHR = 1.10, 95% CI: 1.00–1.22).
Magadi (2023), UK [53]	93,196	Social deprivation was based on the IMD calculated at neighbourhood level and grouped by quintiles (1 = least deprived, 5 = most deprived).	Quintile 1 (least deprived): 16084 (17.3%) 2: 17985 (19.3%) 3: 18735 (20.1%) 4:19,520 (20.9%) 5 (most deprived): 20872 (22.4%)	30-day all cause mortality from discharge	Logistic regression model adjusted for: age, sex, ethnicity, deprivation, comorbidity score, peak AKI stage, elective or emergency admission and season.	The most deprived patients had a greater likelihood of 30-day mortality than the least deprived patients (OR = 1.09, 95% CI: 1.04–1.15). No significant differences were found between the least deprived and all other quintiles (2: OR = 1.03, 95% CI: 0.97–1.08; 3: OR = 1.02, 95% CI: 0.97–1.08; 4: OR = 1.06, 95% CI: 1.00–1.12).
Peracha (2022), England [58]	250,504	Social deprivation was based on IMD and was grouped into quintiles (1 = least deprived, 5 = most deprived)	1 (least deprived): 44510 (17.8%) 2: 49010 (19.6%) 3: 52261 (20.9%) 4: 50761 (20.3%) 5 (most deprived): 52011 (20.8%) Missing: 1,250 (0.5%)	All-cause in-hospital or 30-day mortality from discharge	Multivariable logistic regression models adjusted for: sex, age, ethnicity, deprivation, admission method, AKI alert warning level, comorbidity score, month of alert, diagnosis group and specialised nephrology services	Compared to the most deprived patients, all other deprivation quintiles had decreased 30-day mortality outcomes (1 [least deprived]: aOR = 0.90, 95% CI: 0.88–0.93; 2: aOR = 0.93, 95% CI: 0.90–0.96; 3: aOR = 0.91, 95% CI: 0.89–0.94; 4: aOR = 0.96, 95% CI: 0.93–0.99).
Phillips (2018), Wales [59]	57,654	WIMD score that divides Wales into geographical units grouped by postal code and weighs various social determinants of health. (1 = most deprived, 100 = least deprived)	Total: 20411 reported (35.4%) 1–25: 9,386 (46.0%) 26–50: 765 (3.7%) 51–75: 4,595 (22.5%) 76–100: 5,665 (27.8%)	90-day mortality	Multivariate Cox proportional hazard models adjusting for: age, and pre-existing CKD	90-day mortality was marginally lower in the most affluent population (HR = 0.999; 95% CI: 0.998–0.999; *p* < 0.001)
Roushani (2022), Canada [62]	271	CIMD and ethnocultural composition based on postal code measuring immigration status and visible minorities who cannot speak either official language. Grouped by quintiles (1 + 2 = most deprived, 5 = least deprived)	Quintiles 1 and 2: 29 (10.7%) 3: 32 (11.8%) 4: 53 (19.6%) 5: 157 (57.9%)	90-day all cause mortality from RRT initiation	Multivariable logistic regression models adjusted for: sex, age, geographic location, ethnicity, diabetes, type of residence, income quintile, social deprivation, baseline serum creatinine and time of initiation of RRT relative to COVID-19 diagnosis	No significant differences in 90-day mortality outcomes were found based on social deprivation (OR = 0.86, 95% CI: 0.419 to 1.782).
Sawhney (2023), UK [63]	41,313	Area-level deprivation determined based on people living in the lowest quintile (of the 2016 SIMD) of Scotland. Patients grouped in the lowest quintile (most deprived) were compared to those from all other quintiles.	Most deprived: 3,210 (7.8%)	Incident CKD defined as eGFR < 15 mL/min/1.73 m2 for 90 days or onset of kidney replacement therapies, mortality at 1- and 3-years and long-term mortality	Univariate and multivariate logistic regression models adjusted for age, sex and comorbidities (cancer, CHD, HF, stroke, AFib, PAD, diabetes, hypertension, liver disease, CPD)	No significant differences in long term kidney failure were found between the most deprived and all other patients (aHR: 1.12, 95% CI: 0.95 to 1.33). The most deprived patients had greater risk of an unscheduled hospitalisation within 1-year since AKI (OR = 1.34, 95% CI: 1.23–1.46) and length of stay (8.3 ± 23.2 days vs. 7.0 ± 19.7 days; RR = 1.28, 95% CI: 1.13–1.45) than all other patients. As well, the most deprived patients had greater likelihood of 1-year mortality (aOR = 1.20, 95% CI: 1.09–1.31, *p* < 0.05) and long-term mortality (aOR = 1.18, 95% CI: 1.13–1.25, *p* < 0.05.
Shah (2020), USA [64]	32,598	Patients’ zip codes and determined neighbourhood socioeconomic status, defined as the percentage of zip code residents living below the federal poverty level.	Least to most deprived: < 13.8%: 22167 (68.0%) 13.8% to < 20%: 5,216 (16.0%) 20% to < 40%: 4,564 (14.0%) > 40%: 326 (1.0%) Missing: 326 (1.0%)	Recovery from AKI defined as discontinuation of dialysis due to recovered kidney function within 12 months of kidney failure	Fine and Gray cumulative incidence models with death as a competing risk and Cox proportional hazard models adjusted for: age, year of dialysis initiation, sex, race/ethnicity, body mass index, neighbourhood poverty, region, comorbidities, laboratory values, nursing home and nephrology care	No significant differences were found in recovery outcomes between the least and most deprived (13.8% to < 20%: aHR = 1.00, 95% CI: 0.95–1.05; 20% to < 40%: aHR = 0.96, 95% CI: 0.90–1.02; > 40%: aHR = 0.83, 95% CI: 0.67–1.02).
Shiao (2020), Taiwan [65]	1,322	Regional economic status as the annual disposable income per capita. Patients were grouped into high and low economic status.	High economic status: 992 (75.0%) Low economic status: 330 (25.0%)	Recovery from AKI is defined as weaning of RRT for at least 7 days before death or within 90 days of discharge, all-cause mortality on the 90th day following discharge.	Fine and Gray competing risk regression models with mortality as a competing risk factor and Cox proportional hazard models adjusted for: economic status, age, sex and comorbidities	Patients with higher economic status had better recovery outcomes than those with low economic status (aHR = 1.42, 95% CI: 1.02–1.98, *p* = 0.037). Days to renal recovery by cumulative hazard also significant (*p* = 0.04). No significant differences in mortality risk was found between patients with high and low economic status (*p* = 0.070).
Walker (2021), UK [70]	3,524	Social deprivation based on the SIMD, grouped and ordered from 1 to 10 (1–3 = most deprived, 8–10 = least deprived).	1–3 (most deprived): 880 (25.0%) 4–7: 1,438 (40.8%) 8–10 (least deprived): 1,148 (32.6%) Missing: 58 (1.6%)	All-cause mortality over a median 3.17 year follow-up from the date of index AKI	Cox proportional hazard models adjusted for: age, sex, diabetes, AKI stage, SIMD, previous MI, PVD, cerebrovascular disease, prescription drug use in 3 months leading up to AKI	Compared to the least deprived patients, the most deprived patients had a greater likelihood of all-cause mortality (aHR = 1.16, 95% CI: 1.04–1.29, *p* = 0.007). Patients in the middle group [4–7] did not have significantly different outcomes than the least deprived group (aHR = 1.05, 95% CI: 0.96–1.15, *p* = 0.227).
Wainstein (2023), Multiple [69]	11,138	Patients were grouped per their country’s income based on World Bank classification of LLIMC, UMIC & HIC.	LLMIC: 2,789 UMIC: 704 HIC: 7,645	Length of stay in hospital until discharge or study censor	Logistic regression models and descriptive statistics to assess the relationship between AKI, country income level and in-hospital death adjusted for: age, sex and socioeconomic status, mechanical ventilation, and clinical observations on admission.	Patients from higher income countries had longer median hospital stays (HIC: 20 days, IQR: 11–33; UMIC: 18.5 days, IQR: 11–30; LLMIC: 8 days, IQR: 5–13).

AFib: atrial fibrillation; AKI: acute kidney injury; CI: confidence interval; CIMD: Canadian Index of Multiple Deprivation; CHD: congenital heart disease; CKD: chronic kidney disease; CPD: cardiopulmonary disease; eGFR: estimated glomerular filtration rate; ESKD: end-stage kidney disease; HIC: high income country; HF: heart failure; aHR/HR: adjusted/hazard ratio; ICU: intensive care unit; IMD: Index of Multiple Deprivation; IQR: interquartile range; LLMIC: low- to low-middle income country; MI: myocardial infarction; NHS: National Health Services; aOR/OR: adjusted/odds ratio; PAD: peripheral artery disease; PVD: peripheral vascular disease; RR: relative risk; RRT: renal replacement therapy; SIMD: Scottish Index of Multiple Deprivation; UMIC: upper-middle income country; WIMD = Welsh Index of Multiple Deprivation

**Table 5:** Characteristics & results of studies reporting outcomes stratified by smoking/mental health/housing/geography/insurance *n* = 9

Reference, Country	Sample Size with AKI	Patient Characteristics		Outcome Measure	Analysis	Main findings
		Characteristic of interest	Number of patients (n/%)			
Balogun (2017), USA [36]	11,425	Major depression identified using ICD-9 codes (296.2–296.3) and grouped as with or without depression.	With depression: 8,906 (77.9%)	Recovery from AKI defined as partial or complete recovery within 90 days of AKI event; cardiovascular outcomes defined as readmission for MI, stroke, transient ischemic attack or HF over 90 days since AKI event; all-cause mortality	Multivariate and Cox regression models adjusted for: age, race, baseline eGFR, primary diagnosis at admission, renal recovery status, smoking and Charlson index score	Depression was not found to be a risk factor for partial or complete renal recovery, respectively (aOR = 1.02, 95% CI: 0.92–1.23; aOR = 1.09, 95% CI: 0.93–1.26). However, patients with depression were associated with worse cardiovascular outcomes (aHR = 1.34, 95% CI: 1.23–1.45, *p* < 0.001). Depression was also an independent risk factor for all-cause mortality (aHR = 1.23, 95% CI: 1.12–1.34, *p* < 0.001).
Chen (2023), USA [37]	2,984	Smoking status was collected from a self-reported questionnaire and categorised as never smokers, former smokers and current smokers.	Never smoker: 1,168 (39.1%), Former smoker: 982 (32.9%), Current smoker: 834 (28.0%)	All-cause mortality occurring during hospitalisation with AKI or within 30 days from discharge	Multivariable cause-specific hazards model adjusted for: age, gender, race, study centre, education, BMI, drinking status, comorbidities, medications and clinical status	Compared to patients that never smoked, former smokers were at greater risk of mortality after AKI (aHR = 1.24, 95% CI: 1.02–1.52, *p* < 0.05). Current smokers had a 2-fold increase in AKI-related mortality (aHR = 2.56, 95% CI: 2.09–3.14, *p* < 0.05).
Fan (2019), China [39]	167	Healthcare insurance coverage was collected from medical records and billing information database and grouped as insured or uninsured	Insured: 95 (56.9%) Uninsured: 72 (43.1%)	Hospital and ICU length of stay and ICU mortality	Multivariate logistic regression and descriptive statistics	While insured and uninsured patients had similar hospital lengths of stay (19 days, IQR: 6–28 vs. 16 days, IQR: 4–27), ICU stays were longer in insured patients (12 days, IQR: 4–16 vs. 8 days, IQR: 2–14, *p* < 0.001). Uninsured patients were found to have greater ICU mortality than insured patients (OR = 3.16, 95% CI: 1.50–7.10, *p* = 0.01).
Griffin (2023), USA [42]	182,683	Dementia was collected through inpatient and outpatient claims 12 months prior to admission for AKI	10,691 (5.85%)	1-year mortality	Multivariable analysis using bootstrap resampling to find an appropriate non-parsimonious model adjusting for 30 variables including: Black race, comorbidities, inpatient lab variables, AKI stage	Patients with dementia were associated with increased likelihood of mortality within 1-year of AKI (aOR = 1.91, 95% CI: 1.77–2.07, *p* < 0.0001).
Jensen (2023), Denmark [47]	58,356	Geography was categorised by municipality rurality according to the number of residents and availability of jobs in each of the 5 regions (Capital, Metropolitan, Provincial, Commuter, Rural).	Capital (urban): 14236 (24.4%) Metropolitan: 6,336 (10.9%) Provincial: 13490 (23.1%) Commuter: 10001 (17.1%) Rural: 14293 (24.5%)	Incident CKD defined as outpatient eGFR measurements of < 60 mL/min/1.73 m2 separated by more than 90 days, a hospital diagnosis or procedural code, dependency of dialysis or kidney transplantation; 1-year all-cause mortality	Cox regression models adjusted for: demographics, comorbidities, medication use, lifestyle and social factors, and baseline kidney function	Compared to patients living in the most urban (Capital) region, only patients living in Rural areas were more likely to develop CKD after AKI (aHR = 1.15, 95% CI: 1.05–1.26). Mortality outcomes were not found to be significantly different according to patient geography and rurality (Rural: aHR = 1.02, 95% CI: 0.98–1.07; Commuter: aHR = 1.01, 95% CI: 0.96–1.07; Provincial: aHR = 0.99, 95% CI: 0.95–1.04; Metro: aHR = 0.96, 95% CI: 0.90–1.02).
Mohammed (2018), India [56]	528	Current smoking status was collected from case record forms.	Smoker: 91 (17.2%)	Incident CKD, recovery from AKI and mortality	Descriptive statistics only	Current smokers had greater incidence of CKD following AKI (7.7% vs. 3.0%) and greater incidence of mortality (13.2% vs. 8.9%) than non-smokers. Over half of smokers recovered completely from AKI compared to a third of non-smokers (57.1% vs. 34.3%).
Kolhe (2020), UK [49]	724	Dementia was collected from hospital electronic patient records. Housing was recorded as a care home as the primary residence.	Dementia: 94 (13.0%) Care home residence: 114 (15.7%)	All-cause mortality	Univariate and multivariable logistic regression adjusted for: age, gender, care home residence, comorbidities, COVID-19 status, AKI characteristics and treatments	Dementia was found to be a risk factor for all-cause mortality after AKI (aOR = 2.17, 95% CI: 1.19–3.97, *p* = 0.012). Care home residence was not determined to be a risk factor for mortality (aOR = 0.79, 95% CI: 0.45–1.40, *p* = 0.43).
Roushani (2022), Canada [62]	271	Housing was measured using the ORN COVID-19 data collection tool to group patients into private residences or other. Geography categorised using postal codes into Greater Toronto Area (GTA; urban) & outside GTA (suburban/rural)	GTA (urban): 215 (79.3%) Outside GTA (suburban/rural): 56 (20.7%) Private residence: 250 (92.3%) Other residence: 21 (7.7%)	90-day all cause mortality from RRT initiation	Multivariable logistic regression models adjusted for: sex, age, geographic location, ethnicity, diabetes, type of residence, income quintile, deprivation, baseline serum creatinine and time of initiation of RRT relative to COVID-19 diagnosis	Geography was not found to be a risk factor for 90-day mortality (aOR = 1.06, 95% CI: 0.52–2.18). Similarly, patients living in other non-private residences did not have significantly different mortality outcomes (aOR = 0.42, 95% CI: 0.16–1.14).
Sykes (2019), UK [66]	643	Smoking status was self-reported by patients via structured questionnaires and grouped into current and former smokers.	Not reported for AKI patients.	Progressive CKD defined as the initiation of chronic RRT and all-cause mortality after an index or subsequent AKI events	Competing risk models adjusted for: age, hospital, gender, smoking status, alcohol intake, diabetes, cardiovascular comorbidities and primary renal disease	No significant difference in progression of CKD was found between current and former smokers after the index AKI event (HR = 1.19, 95% CI: 0.90–1.58, *p* = 0.23) or subsequent event (HR = 1.20, 95% CI: 0.56–2.57, *p* = 0.64). Similarly, smoking was not a significant risk factor for mortality after the index AKI event (HR = 1.25, 95% CI: 0.76–2.06, *p* = 0.39) or subsequent event (HR = 1.03, 95% CI: 0.47–2.27, *p* = 0.94).

AKI: acute kidney injury; BMI: body mass index; CI: confidence interval; CKD: chronic kidney disease; eGFR: estimated glomerular filtration rate; GTA: Greater Toronto Area; HF: heart failure; aHR/HR: adjusted/hazard ratio; ICD: International Classification of Diseases; ICU: intensive care unit; IQR: interquartile range; MI: myocardial infarction; aOR/OR: adjusted/odds ratio; RRT: renal replacement therapy

### Sex/gender

**Fig. 5:**
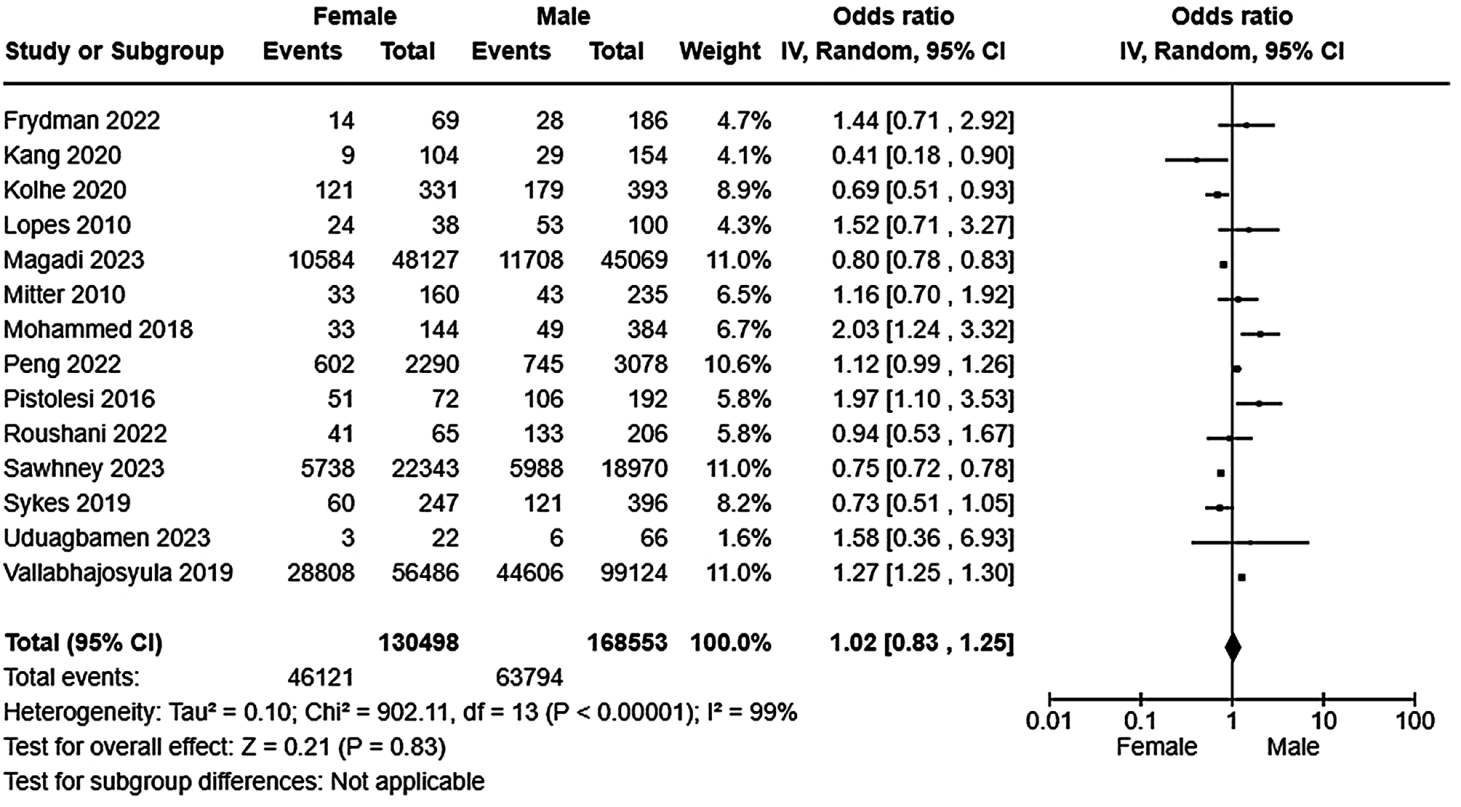
Forest plot of a random effects meta-analysis of all-cause mortality following at least one episode of AKI by sex/gender

**Fig. 6:**
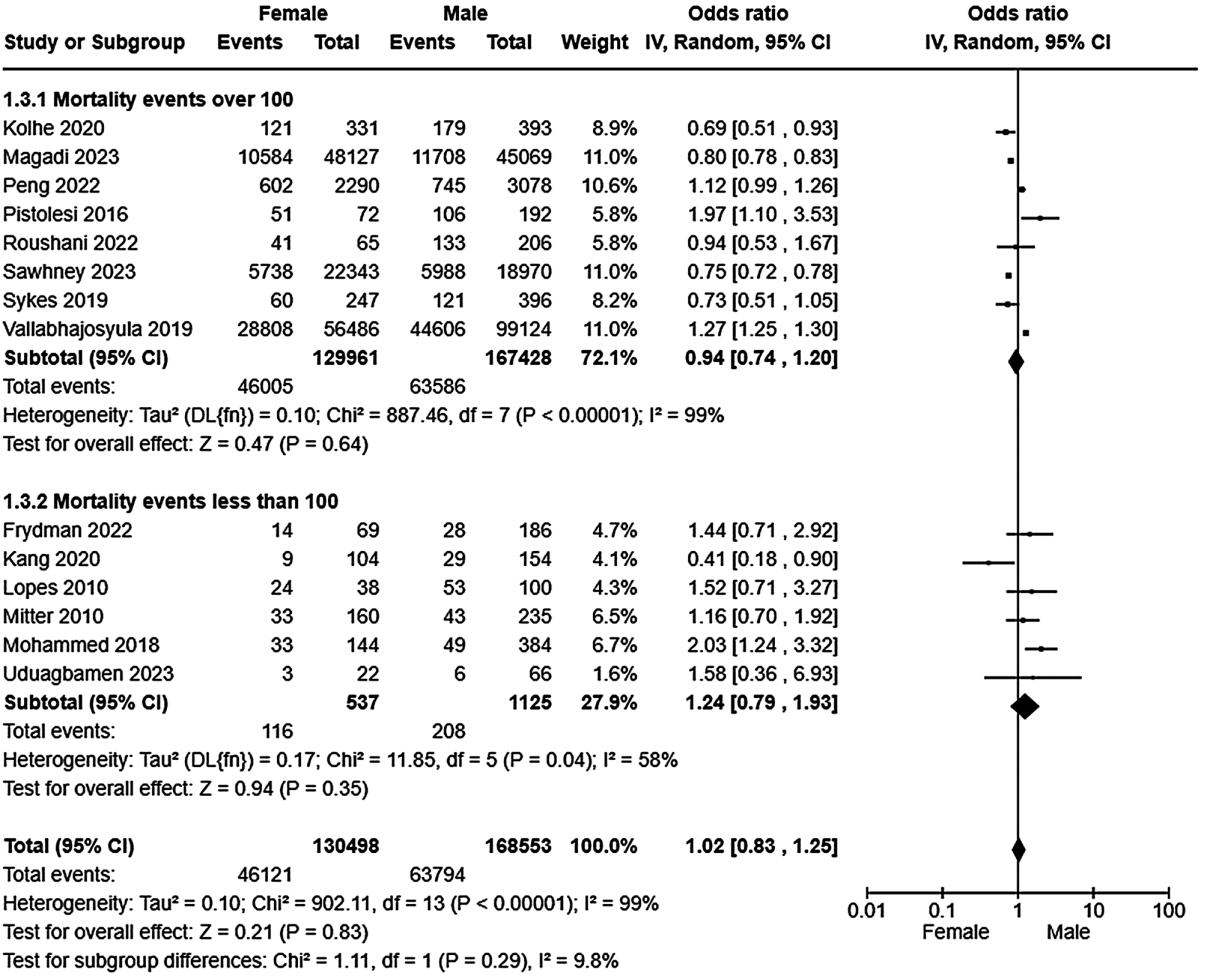
Forest plot of a random effects meta-analysis of all-cause mortality following at least one episode of AKI by sex/gender with subgroup analysis by study size

**Fig. 7:**
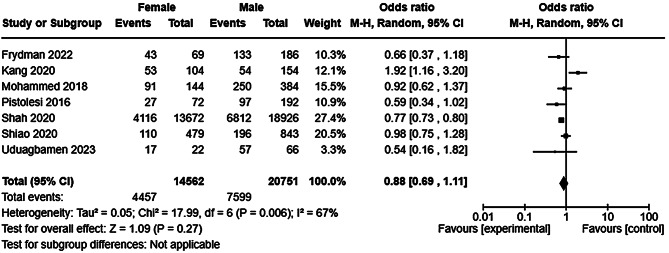
Forest plot of a random effects meta-analysis of AKI recovery following at least one episode of AKI by sex/gender

Detailed characteristics including key results of studies (*n* = 25) reporting sex/gender specific outcomes are listed in Table 2. Twenty studies reported all-cause mortality (see Table 2) of which 14 [41, 48, 49, 52, 53, 55–57, 60, 62, 63, 66–68] were included in a random effects meta-analysis which found in no difference in mortality between males and females (Fig. 5) with very high heterogeneity. Sensitivity analysis including only studies at low risk of bias [52, 53, 55–57, 62, 63] did not alter the findings of the primary synthesis (Supplementary Figure S1). Subgroup analysis by study size partially reduced the observed heterogeneity among the smaller studies suggesting that study size moderates heterogeneity, but did not substantially alter the observed result (Fig. 6). A second subgroup analysis by risk of bias marginally reduced the observed heterogeneity (Supplementary Figure S2), suggesting that study quality moderates, but does not fully explain, heterogeneity. Funnel plot analysis was not suggestive of reporting bias (Supplementary Figure S3). Six studies reported all-cause mortality by sex among patients with AKI, but could not meta-analysed due to an absence of event rate reporting [40, 43, 50, 58, 70, 71]. Four studies reported lower mortality among females [40, 58, 70, 71], one no difference [43], and one higher mortality among females [50]. Seven studies reported recovery from AKI (see Table 2). There was no difference in recovery events by sex/gender with moderate heterogeneity (Fig. 7) which was likely explained in part by variation in the outcome definition employed to measure AKI recovery (see Supplementary Table S2 for study level definitions of AKI recovery, incident and progressive CKD).

Three studies reported incident CKD stratified by sex following AKI [56, 61, 63]. The largest reported no sex-specific difference in long-term kidney failure [63]. One reported a three-fold excess in de novo CKD amongst females with AKI post-ICU admission [61]. A single centre study reported an eight-fold increase in CKD among males, but the timing of follow up was not reported [56].

Two prospective single centre cohort studies reported progressive CKD stratified by sex [46, 66]. No sex-specific differences were reported in progression to kidney failure amongst outpatients with pre-existing CKD post-AKI [66]. One small study reported an excess of CKD progression at 3 years among males [46]. Two studies of hospital inpatients assessed cardiovascular events by sex among subgroups with AKI [41, 55] one of which reported sex-specific differences in stroke in descriptive analysis [55]. One study reported hospitalisation by sex reporting a modest increase in re-hospitalisation for AKI reporting among males [51]. Hospital length of stay was reported by two studies [67, 68]. One reported a slightly shorter length of stay among females, but the results were unadjusted [68]. The other reported no sex-specific differences [67].

### Race/ethnicity

Detailed characteristics including key results of studies (*n* = 14) reporting race/ethnicity specific outcomes are listed in Table 3. There was marked heterogeneity in the reporting of race/ethnicity categories which, combined with an absence of event reporting, precluded meta-analyses. Twelve studies reported all-cause mortality among patients with AKI stratified by race/ethnicity (see Table 3). Six reported lower mortality among patients of Black race compared to other racial or ethnic groups [40, 42, 42, 50, 51, 58]. Three of these studies also reported lower mortality among Asian or South Asian patients compared to white patients [50, 53, 58]. Six studies reported no difference in mortality among different racial/ethnic groups [38, 43, 49, 52, 54, 62].

One study reported AKI recovery stratified by race/ethnicity. Among adults with kidney failure due to AKI, kidney recovery was reported to be lower among those identified as Black, Asian, Hispanic or Native American than white patients [64]. Only one study reported cardiovascular events and ICU admission stratified by race/ethnicity among AKI patients and found no differences between Black and non-Black adults [38]. One study reported hospitalisation events by race/ethnicity in adults with AKI finding a modest increase in rehospitalisation for AKI among Black/African American and Hispanic patients compared to white patients [51]. Two studies reported hospital length of stay between Black and white patients [44, 54]. One reported no difference [54] while another reported a modest increase among white patients, however, the results were descriptive [44].

### Socioeconomic deprivation

Eleven studies reported outcomes in patients with AKI stratified by socioeconomic deprivation as defined by the included studies (Table 4). A meta-analysis was not performed due to differences in classification of deprivation. Nine studies (see Table 4) reported mortality among patients with AKI. Six showed an increase in all-cause mortality among the most deprived sub-populations compared to the most affluent with relative effect sizes varying from minimal (i.e. HR 0.999 least vs most deprived) to modest (i.e. HR 1.20 most vs least deprived) [45, 58, 59, 63, 70, 71]. Three studies reported no difference in mortality [53, 62, 65].

Three studies reported differences in recovery from AKI although variable definitions were employed (see Supplementary Table S2) [64, 65, 71]. One showed greater recovery in those of high compared to low socioeconomic status among critically ill patients requiring KRT [65]. Two studies did not show any significant difference [64, 71]. Two studies reported hospital length of stay [63, 69]. Sawhney et al. [63] reported increased length of stay among the most deprived while Wainstein et al. [69] among the most affluent. Sawhney et al. [63] also reported increased hospitalization post AKI amongst the most deprived, but no difference in incident CKD by socioeconomic status.

### Other comparators of interest

Three studies discussed AKI outcomes stratified by smoking status all of which reported mortality [37, 56, 66]. Two reported increased mortality in current smokers [37, 56], while one did not report a significant difference [66]. Mohammed et al. [56] also reported increased AKI recovery and incident CKD among smokers. Sykes [66] did not report a significant difference in progressive CKD by smoking status.

Three studies reported AKI outcomes stratified by mental health conditions of interest [36, 42, 49]. Balugon et al. [36] showed an increase in cardiovascular events and mortality among patients with major depression. Two studies [42, 49] showed a two-fold relative increase in mortality among hospitalised patients with dementia following an episode of AKI compared to patients without dementia. No studies compared outcomes based on comorbid substance use or bipolar disorder.

Three studies reported AKI outcomes stratified by housing or geography, none of which reported any differences in all-cause mortality by comparators [47, 49, 62], however, one reported a modest increase in incident CKD among a rural compared to a metropolitan sub-population [47]. Only one study reported AKI outcomes stratified by insurance status reporting no difference in hospital length of stay by insurance status, but higher mortality among uninsured patients [39].

No studies were identified which reported stratified results or subgroup analysis by income, education or employment.

### Risk of bias

Summary risk of bias assessment for all studies is detailed in Supplementary Table S3. Overall, twenty six studies were rated as ‘*good’* [36–39, 42–47, 51–59, 61–65, 70, 71], eight ‘*poor’* [40, 41, 49, 50, 60, 67–69] and two ‘*fair’* quality [48, 66]. Frequent sources of bias included selection bias and outcome misclassification with the former relating primarily to the representativeness of the exposed cohort (e.g. a selected group of adults with multiple exclusion criteria applied creating a material risk of selection bias).

## Discussion

This review identified a paucity of studies reporting data on health inequalities and outcomes among AKI patients. Evidence predominantly related to the impact of sex/gender, race/ethnicity and socioeconomic deprivation. No studies were identified from low-income countries with only three containing data from lower-middle income countries. There was a lack of evidence pertaining to the impact of mental health conditions, healthcare insurance, housing, geography and smoking status and no reports quantifying the impact of income, education, employment or substance use. On pooling relevant studies, no sex/gender-specific differences in all-cause mortality or AKI recovery were observed. Half of studies reported variation of mortality by race/ethnicity while socioeconomic deprivation was found to be an independent predictor of mortality in most relevant studies. The findings from other comparators and outcomes were based on a small number of individual studies (i.e. three or fewer per outcome).

Pre-clinical studies have demonstrated that sex-specific differences in AKI vary with aetiology and age reflecting sex hormonal influences at pre- and post-menopause [73]. Females benefit from an oestrogen derived protection against ischaemia-reperfusion injury [74] and nephrotoxin induced AKI in animal models particularly at younger ages [73, 75]. These findings are supported by evidence from human studies highlighting that women have a lower risk of community [76] and hospital-acquired AKI [77, 78], sepsis-associated AKI [79] and AKI requiring dialysis [80], despite the KDIGO 2012 guideline citing female sex as a susceptibility factor for AKI [24]. However, conflicting results have been reported by aetiology, with cardiac surgery associated AKI and contrast induced AKI showing no such sex differences [77, 81]. This meta-analysis contains patients across a range of ages and aetiologies with variation in the timing of outcome ascertainment which may partially explain the lack of sex-specific variation in survival. The synthesis of AKI recovery found no sex-specific differences, however, most studies did not account for the competing risk of death and variable time-frames were examined. Study level variation in the measurement of AKI recovery as highlighted in this review likely reflects the lack of an international consensus definition.

Disparities in AKI incidence have been reported according to race/ethnicity [81–84]. Definitive conclusions on the impact of race/ethnicity on mortality are challenging due to variability in the definition and measurement of race/ethnicity among studies included in our review with multiple comparisons of varying utility. Research in this subject is hampered by a lack of uniform definition of the terms employed [85]. Race/ethnicity differences have been reported across the spectrum of kidney diseases although underlying mechanisms are a complex interplay of genetic, socio-cultural and environment factors which remain incompletely understood [82, 83, 86–91]. Black patients have been reported to be at modestly increased risk of hospitalisation for AKI [83, 91], but not after adjustment for baseline comorbidities, renal function, renin angiotensin-aldosterone inhibition [91], income or insurance status [83]. These findings highlight the importance of addressing diabetes, hypertension, financial resources and access to healthcare in reducing race related inequalities in AKI [83, 91]. Other potentially contributory factors include high risk genetic alleles (e.g. apolipoprotein L1) [91] in addition to systemic discrimination in healthcare, research and wider society [92]. These factors warrant further study to better understand racial/ethnic differences in AKI outcomes.

The findings related to deprivation reflect a socioeconomic gradient common to many diseases [93, 94] with patients from socioeconomically deprived areas being more likely to die than their affluent counterparts. Health inequalities have been described in relation to other types of kidney disease [4, 5] with socioeconomically deprived patients with CKD reported to have faster progression [95, 96], higher risk of cardiovascular disease [97, 98] and premature mortality [4, 99, 100]. Similar disparities have been reported in the transplant literature [5, 101] with proxy measures of social capital, such as house value [102], car ownership or lack of educational qualifications [103], being predictive of access to living donor kidney transplantation [5, 102, 103]. Effect sizes in studies reporting a socioeconomic gradient in AKI mortality in our review ranged from near negligible [45, 59] to modest in studies comparing low and high socioeconomic groups [45, 58, 59, 63, 70, 71]. Sawhney et al. [63] reported that patients with a range of kidney diseases from deprived areas were more likely to miss scheduled appointments and attend hospital emergently than their affluent counterparts, but less likely to receive community diagnostic monitoring. The factors underlying such discrepancies warrant further study.

The lack of studies from low-income countries is concerning given the differential impact of AKI in resource poor settings [27, 103–106]. Two studies were identified solely from lower-middle income countries [56, 67] based on the world bank data classification [72]. Uduagbamen et al. [67] reported no sex-specific differences in AKI outcomes among adults following major surgery in a single centre from Nigeria, but the study was underpowered to detect subgroup differences. Wainstein et al.’s multinational study [69] included data from five LMIC, but no LICs. The results showed higher in-hospital death among critically ill adults with Covid-19 and AKI in LMIC compared to HICs. The burden of AKI in low and lower-middle income countries is unclear due to a lack of diagnostics, reporting systems and under detection of cases often resulting from prohibitively long travel to regional hospitals [27, 106, 107]. The use of expanded criteria for AKI based on a decrease in serum creatinine has therefore been proposed to capture cases during the recovery phase of AKI to account for late presentation [106, 108]. This extended KDIGO definition improves detection in resource limited settings [109] where AKI is usually community-acquired with a disproportionate impact on working-age adults and children, as a result of infections, diarrhoea, dehydration and traditional medicines [106, 110, 111]. Environmental factors such as poor sanitation, lack of access to clean water, food insecurity and vector-borne diseases are important contributors [27]. Access to care may vary by sex/gender as males may be more likely to obtain medical care due to their social status as household economic provider in settings where cost is implicated. This compares to high-income settings where AKI predominates among older, multimorbid patients with critical illness [106, 110]. Injustice is therefore compounded given that AKI in low income settings is frequently preventable and treatable with a good prospect of recovery [17, 107, 110]. An absence of expertise and equipment required for acute dialysis undoubtedly leads to avoidable mortality [104, 108]. However, as highlighted by this review, there is a lack of AKI outcome data related to these inequities in low-income settings. There is therefore an urgent need for the global nephrology community to collaborate on international population-wide estimates of AKI to inform public health policy.

This study has several strengths. It is the first to our knowledge to synthesise available evidence on outcome comparison by inequalities among adults with AKI. The review utilises real world evidence including a representative case mix of patients with AKI and is therefore generalisable and directly applicable to clinicians and policymakers. We employed a methodologically rigorous and reproducible method to systematically search and synthesise the literature and our approach takes account of the social determinants of health in addition to individual lifestyle risk factors. We adopted an intentionally inclusive approach with regards to secondary outcome measures, such as AKI recovery, which lack a consensus definition, to inform the evidence base in an area which is under studied.

This study also has limitations. These results are based on observational evidence which is subject to residual confounding. Although study size and risk of bias partially explained the observed heterogeneity in the meta-analysis, residual unexplained heterogeneity was apparent and likely explained by clinical and contextual differences in patient case mix. There are inherent limitations in pooling unadjusted mortality estimates from heterogenous observational studies, however, the synthesis reflects limitations in the primary evidence base which is itself a representation of real-world clinical practice. We were unable to test for the impact of country income level due to an absence of evidence from low-income settings. Furthermore, meta-analysis of most included results was not possible due to methodological and statistical heterogeneity. This in part reflects the complex and interdependent nature of health inequalities which are variably measured and may not be mutually exclusive in their impact on outcomes. The findings are based on different geographic regions and models of care, including public and private health systems, and further work is required to understand AKI incidence across such variable settings, particularly in low-income settings. Due to insufficient data, it was not possible to determine the impact of baseline CKD by geographic region as a determinant of AKI. Finally, this review does not distinguish between the socio-biological variables of sex and gender or race and ethnicity which are poorly distinguished in primary research [85]. These terms were frequently used interchangeably within the primary studies hence the original terms employed are reported.

## Conclusions

Our systematic review highlights a paucity of evidence related to health inequalities and AKI. Addressing these gaps is important to inform targeted implementation of limited resource and guide appropriate post-hospital follow-up [112–114]. Specifically, incorporating evidence on health inequalities into novel prognostic models, educational activities and ambulatory care management approaches will help to reduce inequitable differences in outcome [21]. These findings meaningfully extend the literature on sex-specific differences in AKI outcomes and highlight the need for follow-up studies of incident CKD, cardiovascular events and re-hospitalisation to inform health policy. Studies are urgently required from low-income settings if progress is to be made towards achieving equitable kidney health. These results support the need for policies to increase resource allocation for patients with AKI who live in socioeconomic deprivation due to an increased risk of mortality. There was also a lack of evidence in relation to mental health conditions, insurance access, housing and geography with no reports quantifying the impact of income, education, employment or substance use. These factors warrant exploration through further studies to identify high-risk groups who may benefit from targeted intervention. Increasing understanding of inequalities in AKI will inform policy and practice with the aim of achieving equitable healthcare systems which serve all people fairly.

## Electronic supplementary material

Below is the link to the electronic supplementary material.

Supplementary Material 1

## Data Availability

The consensus data extraction forms, risk of bias assessments and summary statistics (i.e. Microsoft excel spreadsheets) supporting the findings of this study are available upon request to the corresponding author (SB).

## Abbreviations

AKD: Acute kidney disease
AKI: Acute kidney injury
CKD: Chronic kidney disease
ESKD: End stage kidney disease
ICU: Intensive care unit
KRT: Kidney replacement treatment
LIC: Low income countries
LMIC: Lower-middle income
PRISMA: Preferred reporting items for systematic reviews and meta-analyses
SDOH: Social determinants of health
