# Supplementary material for: Health inequalities and outcomes following acute kidney injury: a systematic review & meta-analyses of observational studies
Source: BMC Nephrol. 2025 Aug 27;26:494. doi: 10.1186/s12882-025-04391-x (PMC12382016; doi:10.1186/s12882-025-04391-x)

**Supplementary Information S1: Full details of inclusion & exclusion criteria according to PICO**

*Population*

Include:

- Studies exclusively in the adult population (ie all participants >/=16 years old)

Exclude:

- paediatric population (< 16 years)
- patients with treated ESKD (ie receiving kidney replacement treatment via dialysis or transplantation)

*Exposure*

Include:

- at least one episode of acute kidney injury (determined by elevation in serum creatinine or initiation of acute renal replacement therapy (haemodialysis, haemofiltration or peritoneal dialysis) for AKI as per consensus guidelines [ie KDIGO / RIFLE / AKIN] and/or administrative coding)

Exclude:

- no clear definition of AKI provided
- definition of AKI not via consensus guidelines or administrative coding

*Comparison(s)*

Include:

Subgroup analysis or stratification of results according to any of the following sociodemographic factors:

- sex/gender
- race/ethnicity
- socioeconomic deprivation
- income
- education
- employment
- housing
- smoking status
- mental health conditions (ie dementia, depression, substance use disorders or bipolar disorder)
- geography (rural vs urban)
- healthcare insurance status

Exclude:

- Does not report subgroup analysis or stratification of results according to any of the listed comparators of interest

*Outcome*

Include:

- Reports any of the outcomes listed below, and as defined by the studies themselves, following an episode of AKI
  - progression to acute kidney disease (AKD)
  - incident chronic kidney disease (CKD)
  - progressive CKD
  - recovery from AKI
  - cardiovascular events
  - hospitalizations or re-hospitalizations
  - ICU admission
  - length of stay
  - all-cause mortality

Exclude:

- Does not report any of the listed outcomes of interest following an episode of AKI

*Study design / reporting*

Include:

- Primary observational studies

Exclude:

- Interventional studies
- Non-primary observational studies including review articles
- Conference abstracts or proceedings
- Non-English language studies
- Full text not available

**Supplementary Information S2a: Newcastle-Ottawa Quality Assessment Scale for Cohort studies**

**Selection - Cohort** (maximum one star for each numbered item)

1) Representativeness of the exposed cohort

a) truly representative of the average adult (>/= 16 years) in a community or hospital setting (eg population based or multi-centre & random sampling) *

b) somewhat representative of the average adult (>/= 16 years) in a community or hospital setting (eg single-centre & non-random sampling) *

c) selected group of adults (>/= 16 years) (eg non-consecutive series of cases)

d) no description of the derivation of the cohort / unclear

2) Selection of the non exposed cohort

a) drawn from the same setting as the exposed cohort *

b) drawn from a different source

c) no description of the derivation of the non-exposed cohort / unclear

3) Ascertainment of exposure

a) secure record (eg medical records to determine AKI) *

b) administrative database (eg ICD diagnostic codes) *

c) written self report or other non-secure record

d) no description / unclear

4) Demonstration that outcome of interest was not present at start of study

a) yes *

b) no

c) no description / unclear

**Comparability - Cohort** (maximum two stars)

1) Comparability of cohorts on the basis of the design or analysis

a) study controls for age (the most important factor) & any additional factor **

b) study controls for age *

c) study controls for any additional factor *

d) study does not control for any of the above factors

**Outcome - Cohort** (maximum one star for each numbered item)

1) Assessment of outcome

a) independent blind assessment or confirmation of the outcome by reference to secure primary records (ie medical records) *

b) record linkage (e.g. identified through ICD codes on database records) *

c) self report or other determination (ie no reference to original medical records to confirm the outcome)

d) no description / unclear

2) Was follow-up long enough for outcomes to occur

a) yes (ie 7 days) *

b) no

c) no description / unclear

3) Adequacy of follow up of cohorts

a) complete follow up - all subjects accounted for *

b) subjects lost to follow up unlikely to introduce bias (ie < 20% loss to follow up) or adequate description provided of those lost *

c) follow up rate < 80% & no description of those lost

d) no statement

**Supplementary Information S2b: Newcastle-Ottawa Quality Assessment Scale for Case-control studies**

**Selection – Case-control** (maximum one star for each numbered item)

1) Is the case definition adequate?

a) yes, with independent validation eg by reference to secure primary records ie medical records *

b) yes, eg record linkage identified through ICD codes on database records or death records *

c) no, eg self-reported

d) no description / unclear

2) Representativeness of the cases

a) consecutive or obviously representative series of cases *

b) potential for selection biases or not stated

c) no description / unclear

3) Selection of Controls

a) community controls if community cases or hospital controls if hospital cases *

b) controls derived from a different setting (eg community controls matched to hospital cases)

c) no description / unclear

4) Definition of Controls

a) no history of disease (endpoint) *

b) no description / unclear

**Comparability – Case-control** (maximum two stars)

1) Comparability of cases and controls on the basis of the design or analysis

a) study controls for age (the most important factor) & any additional factor **

b) study controls for age *

c) study controls for any additional factor *

d) study does not control for any of the above factors

**Exposure – Case-control** (maximum one star for each numbered item)

1) Ascertainment of exposure

a) secure record (eg medical records to determine AKI) *

b) administrative database (eg ICD diagnostic codes) *

c) written self report or other non-secure record

d) no description / unclear

2) Same method of ascertainment for cases and controls

a) yes *

b) no

c) no description / unclear

3) Non-Response rate

a) same rate for both groups *

b) non respondents described

c) rate different

d) no description / unclear

**Supplementary Information S2c: Newcastle-Ottawa Quality Assessment Scale for Cross-sectional studies**

**Selection – Cross-sectional** (maximum one star for each numbered item)

1) Representativeness of exposed subjects

a) truly representative of the average adult (>/= 16 years) in a community or hospital setting (eg population based or multi-centre & random sampling) *

b) somewhat representative of the average adult (>/= 16 years) in a community or hospital setting (eg single-centre & non-random sampling) *

c) selected group of adults (>/= 16 years) (eg non-consecutive series of cases)

d) no description of the derivation of the cohort / unclear

2) Sample size

a) justified & satisfactory (ie power calculation) *

b) less than 30 participants assessed for outcome

c) no description / unclear

3) Non-respondents

a) proportion of the target sample recruited is satisfactory (ie >80%) or a comparative summary of non-respondents is provided *

b) proportion of the target sample recruited is unsatisfactory (ie < 80%) & no summary data provided on non-respondents

c) no description / unclear

4) Ascertainment of exposure

a) secure record (eg medical records to determine AKI) *

b) administrative database (eg ICD diagnostic codes) *

c) written self report or other non-secure record

d) no description / unclear

**Comparability – Cross-sectional** (maximum two stars)

1) Comparability of cohorts on the basis of the design or analysis

a) study controls for age (the most important factor) & any additional factor **

b) study controls for age *

c) study controls for any additional factor *

d) study does not control for any of the above factors

**Exposure – Cross-sectional** (maximum two stars for assessment of outcome & one star for statistical tests)

1) Assessment of outcome

a) independent blind assessment or confirmation of the outcome by reference to secure primary records (ie medical records) **

b) record linkage (e.g. identified through ICD codes on database records) **

c) self-report or other determination (ie no reference to original medical records to confirm the outcome)

d) no description / unclear

2) Statistical tests

a) the statistical test used to analyze the association is clearly described and appropriate, and the measurement is presented as either an OR, CI and P value or a beta coefficient, SE and P value *

b) the statistical test is inappropriate, poorly described, or incomplete

c) no description / unclear

**Supplementary Information S2d: Newcastle-Ottawa Overall Quality Assessment**

**Overall quality**

Good quality: 3 or 4 stars in selection domain AND 1 or 2 stars in comparability domain AND 2 or 3 stars in outcome/exposure domain

Fair quality: 2 stars in selection domain AND 1 or 2 stars in comparability domain AND 2 or 3 stars in outcome/exposure domain

Poor quality: 0 or 1 star in selection domain OR 0 stars in comparability domain OR 0 or 1 stars in outcome/exposure domain

**Supplementary Table S1: Electronic database search strategy**

| **MEDLINE:** | 1. *Acute Kidney Injury/ OR Kidney Tubular Necrosis, Acute/ OR ((acute kidney or acute renal) adj2 (injur* or insufficien* or failure* or replacement therap*)).tw. OR "acute kidney tubular necrosis".tw. OR “acute renal tubular necrosis”.tw. OR AKI.tw. OR "Acute RRT".tw. OR AKI-D.tw. OR "acute h?emodialysis".tw. OR "acute h?emofiltration".tw. OR "acute h?emodiafiltration".tw. OR "continuous RRT".tw. OR "continuous renal replacement therap*".tw. OR "acute peritoneal dialysis".tw.  AND  2. Mental Health/ OR Mental Disorders/ OR Substance-Related Disorders/ OR exp Geography, Medical/ OR Ethnicity/ OR Race Factors/ OR “Health Disparate, Minority and Vulnerable Populations”/ OR Minority Health/ OR Socioeconomic Factors/ OR Health Insurance/ OR *Sex Factors/ OR Gender Equity/ OR Health Inequities/ OR Social Determinants of Health/ OR (“substance-related disorder*” OR “drug abuse” OR “mental health” OR “mental disorder*” OR tobacco OR smok* OR depress* OR dementia OR bipolar OR “substance abuse” OR inequal* OR inequit* OR depriv* OR rural* OR urban* OR remote OR ethnic* OR race OR racial OR black* OR white* OR asian* OR caucasian* OR hispanic* OR african* OR indigenous OR aboriginal* OR “minority population*” OR “minority group*” OR socioeconomic* OR income OR poverty OR “*economic status” OR insurance OR “*insured” OR medicaid OR “social class” OR “social support” OR “access to health care” OR gender OR “sexual orientation” OR “social determinant*” OR education OR housing OR “housing stability” OR employment).tw. OR ethnology.sh.  AND  3. Epidemiologic studies/ OR Exp case control studies/ OR Exp cohort studies/ OR Case control.tw. OR (cohort adj (study or studies)).tw. OR Cohort analy*.tw. OR (Follow up adj (study or studies)).tw. OR (observational adj (study or studies)).tw. OR Longitudinal.tw. OR Retrospective.tw. |
| --- | --- |
| **Embase** | 1. *Acute Kidney Failure/ OR Acute Kidney Tubule Necrosis/ OR ((acute kidney or acute renal) adj2 (injur* or insufficien* or failure* or replacement therap*)).tw. OR "acute kidney tubular necrosis".tw. OR “acute renal tubular necrosis”.tw. OR AKI.tw. OR "Acute RRT".tw. OR AKI-D.tw. OR "acute h?emodialysis".tw. OR "acute h?emofiltration".tw. OR "acute h?emodiafiltration".tw. OR "continuous RRT".tw. OR "continuous renal replacement therap*".tw. OR "acute peritoneal dialysis".tw.  AND  2. Mental Health/ OR Mental Disease/ OR Drug Dependence/ OR exp Medical Geography/ OR Ethnicity/ OR Race/ OR Health Disparity/ OR Minority Group/ OR Vulnerable Population/ OR Minority Health/ OR Socioeconomics/ OR Health Insurance/ OR *Sex Difference/ OR Gender Inequality/ OR “Social Determinants of Health”/ OR (“substance-related disorder*” OR “drug abuse” OR “mental health” OR “mental disorder*” OR tobacco OR smok* OR depress* OR dementia OR bipolar OR “substance abuse” OR inequal* OR inequit* OR depriv* OR rural* OR urban* OR remote OR ethnic* OR race OR racial OR black* OR white* OR asian* OR caucasian* OR hispanic* OR african* OR indigenous OR aboriginal* OR “minority population*” OR “minority group*” OR socioeconomic* OR Income OR Poverty OR “*economic status” OR insurance OR “*insured” OR medicaid OR “social class” OR “social support” OR “access to health care” OR Gender OR “sexual orientation” OR “social determinant*” OR education OR housing OR “housing stability” OR employment).tw. OR ethnology.sh.  AND  3. Clinical study/ OR Case control study/ OR Family study/ OR Longitudinal study/ OR Retrospective study/ OR (Prospective study/ NOT Randomized controlled trials/) OR Cohort analysis/ OR (Cohort adj (study or studies)).mp. OR (Case control adj (study or studies)).tw. OR (follow up adj (study or studies)).tw. OR (observational adj (study or studies)).tw. OR (epidemiologic$ adj (study or studies)).tw. |
| **Web of Science Core Collection** | 1. (TI=(((acute kidney) NEAR/2 (injur* or insufficien* or failure* or replacement)) OR ((acute renal) NEAR/2 (injur* or insufficien* or failure* or replacement)) OR "acute kidney tubular necrosis" OR AKI OR "Acute RRT" OR AKI-D OR "acute h$emodialysis" OR "acute h$emofiltration" OR "acute h$emodiafiltration" OR "continuous RRT" OR "continuous renal replacement therap*" OR "acute peritoneal dialysis") OR AB=(((acute kidney) NEAR/2 (injur* or insufficien* or failure* or replacement)) OR ((acute renal) NEAR/2 (injur* or insufficien* or failure* or replacement)) OR "acute kidney tubular necrosis" OR AKI OR "Acute RRT" OR AKI-D OR "acute h$emodialysis" OR "acute h$emofiltration" OR "acute h$emodiafiltration" OR "continuous RRT" OR "continuous renal replacement therap*" OR "acute peritoneal dialysis"))  AND  2. (TI=(“substance-related disorder*” OR “drug abuse” OR “mental health” OR “mental disorder*” OR tobacco OR smok* OR depress* OR dementia OR bipolar OR “substance abuse” OR inequal* OR inequit* OR depriv* OR rural* OR urban* OR remote OR ethnic* OR race OR racial OR black* OR white* OR asian* OR caucasian* OR hispanic* OR african* OR indigenous OR aboriginal* OR “minority population*” OR “minority group*” OR socioeconomic* OR income OR poverty OR “economic status” OR insurance OR “insured” OR “uninsured” OR medicaid OR “social class” OR “social support” OR “access to health care” OR gender OR “sexual orientation” OR “social determinant*” OR education OR housing OR “housing stability” OR employment) OR AB=(“substance-related disorder*” OR “drug abuse” OR “mental health” OR “mental disorder*” OR tobacco OR smok* OR depress* OR dementia OR bipolar OR “substance abuse” OR inequal* OR inequit* OR depriv* OR rural* OR urban* OR remote OR ethnic* OR race OR racial OR black* OR white* OR asian* OR caucasian* OR hispanic* OR african* OR indigenous OR aboriginal* OR “minority population*” OR “minority group*” OR socioeconomic* OR income OR poverty OR “economic status” OR insurance OR “insured” OR “uninsured” OR medicaid OR “social class” OR “social support” OR “access to health care” OR gender OR “sexual orientation” OR “social determinant*” OR education OR housing OR “housing stability” OR employment))  AND  3. (TI=(“Case control” OR (cohort NEAR/1 (study or studies)) OR Cohort analy* OR (Follow up NEAR/1 (study or studies)) OR (observational NEAR/1 (study or studies)) OR Longitudinal OR Retrospective) OR AB=(“Case control” OR (cohort NEAR/1 (study or studies)) OR Cohort analy* OR (Follow up NEAR/1 (study or studies)) OR (observational NEAR/1 (study or studies)) OR Longitudinal OR Retrospective)) |

**Supplementary Table S2: Definitions and measures of incident/progressive CKD or AKI recovery employed by relevant studies by comparators of interest**

| **Author, Year** | **Outcome Measure** | **Comparator(s)** |
| --- | --- | --- |
| ***Incident chronic kidney disease*** | |  |
| Jensen, 2023 | Incident CKD defined as >/= 2 outpatient eGFR measurements of <60 mL/min/1.73m^2^ separated by more than 90 days, a hospital diagnosis or procedural code, dependency of dialysis due to CKD or kidney transplantation | Geography (urban vs rural) |
| Mohammed, 2018 | Incident CKD (definition of outcome measure not reported by primary study) | Sex/gender; smoking status |
| Rimes-Stigare, 2018 | Incident CKD according to KDOQI stages of CKD based on GFR categories at follow-up between 2 and 7 months who survived to 90 days | Sex/gender |
| Sawhney, 2023 | Onset of kidney failure as sustained eGFR <15 mL/min/1.73 m^2^ for at least 90 days on at least two blood tests and all intervening tests or onset of dialysis or transplant | Sex/gender; socioeconomic status |
| ***Progressive chronic kidney disease*** | |  |
| Horne, 2017 | Progressive CKD at 3 years following enrollment defined as a decrease in eGFR (≥25%) associated with a decline in eGFR stage | Sex/gender |
| Sykes, 2019 | Progressive CKD defined as the initiation of chronic RRT (defined as the date of first session of chronic haemodialysis or peritoneal dialysis or date of renal transplant) | Sex/gender; smoking status |
| ***Acute kidney injury recovery*** | |  |
| Balogun, 2017 | Complete recovery as return of SCr to a level <1.25 times baseline SCr and independence from dialysis; partial recovery as SCr >/= 1.25 baseline SCr at end of recovery period; no recovery as dialysis requirement for 90+ days | Mental health (depression) |
| Frydman, 2022 | Any (early + partial) recovery from AKI defined as a return to creatinine level within 0.3ml/dL of that at baseline; early within 72h of AKI diagnosis; partial is improvement of creatinine to a level higher than 0.3mg/dL of baseline within 72h | Sex/gender |
| Hounkpatin, 2020 | Recovery defined by comparing the lowest SCr value within 90 and 180 days to the baseline SCr. Full recovery defined as a return to </=1.2 times, partial a return to >1.2 & <1.5 times & no recovery defined as SCr remaining 1.5 times the baseline SCr | Socioeconomic status |
| Kang 2020 | Failure to recover from AKI defined as still dependent on dialysis or SCr decreasing by less than 25% from the peak concentration | Sex/gender |
| Mohammed, 2018 | Recovery from AKI (definition of outcome measure not reported by primary study) | Sex/gender; smoking status |
| Pistolesi, 2016 | Recovery from AKI defined as independence from RRT | Sex/gender |
| Shah, 2020 | Recovery from AKI defined as discontinuation of dialysis due to recovered kidney function within 12 months of kidney failure | Sex/gender; race/ethnicity; socioeconomic status |
| Shiao, 2020 | Recovery from AKI defined as weaning of RRT for at least 7 days before death or within 90 days of discharge | Sex/gender; socioeconomic status |
| Uduagbamen, 2023 | Complete recovery of kidney function: SCr returns to pre-induction value or less. Partial recovery: reduced severity of AKI grade but not less than stage 1 | Sex/gender |

**Supplementary Table S3: Summary risk of bias assessment for all studies**

| **Study** | **Selection** | **Comparability** | **Outcome/Exposure** | **Overall** |
| --- | --- | --- | --- | --- |
| Balogun 2017 | High | High | High | Good quality |
| Chen 2023 | High | High | High | Good quality |
| Egbuche 2021 | High | Some concerns | High | Good quality |
| Fan 2019 | High | High | Some concerns | Good quality |
| Fisher 2020 | Some concerns | Low | Some concerns | Poor quality |
| Frydman 2022 | Low | Low | Some concerns | Poor quality |
| Griffin 2023 | Some concerns | High | High | Good quality |
| Gupta 2021 | Some concerns | High | High | Good quality |
| Hassan 2021 | Some concerns | High | High | Good quality |
| Holmes 2019 | Some concerns | Some concerns | Some concerns | Good quality |
| Horne 2017 | High | High | High | Good quality |
| Hounkpatin 2020 | High | High | Some concerns | Good quality |
| Jensen 2023 | Some concerns | High | High | Good quality |
| Kang 2020 | Low | Some concerns | High | Fair quality |
| Kolhe 2016 | Some concerns | High | Low | Poor quality |
| Kolhe 2020 | High | High | Low | Poor quality |
| Liu 2019 | Some concerns | High | Some concerns | Good quality |
| Lopes 2010 | Some concerns | Some concerns | High | Good quality |
| Magadi 2023 | Some concerns | High | High | Good quality |
| Mathioudaki 2016 | Some concerns | High | High | Good quality |
| Mitter 2010 | Some concerns | High | High | Good quality |
| Mohammed 2018 | Some concerns | High | Some concerns | Good quality |
| Peng 2022 | Some concerns | High | Some concerns | Good quality |
| Peracha 2022 | High | High | Some concerns | Good quality |
| Phillips 2018 | Some concerns | High | High | Good quality |
| Pistolesi 2016 | Low | High | Low | Poor quality |
| Rimes-Stigare 2018 | High | High | High | Good quality |
| Roushani 2022 | High | High | Some concerns | Good quality |
| Sawhney 2023 | High | High | Some concerns | Good quality |
| Shah 2020 | Some concerns | High | High | Good quality |
| Shiao 2020 | High | High | High | Good quality |
| Sykes 2019 | Low | High | High | Fair quality |
| Uduagbamen 2023 | Low | High | Low | Poor quality |
| Vallabhajosyula 2019 | Some concerns | High | Low | Poor quality |
| Wainstein 2023 | Some concerns | High | Low | Poor quality |
| Walker 2021 | High | High | High | Good quality |

**Supplementary Figure S1: Sensitivity analysis of all-cause mortality following at least one episode of AKI by sex /gender including only studies at low risk of bias**


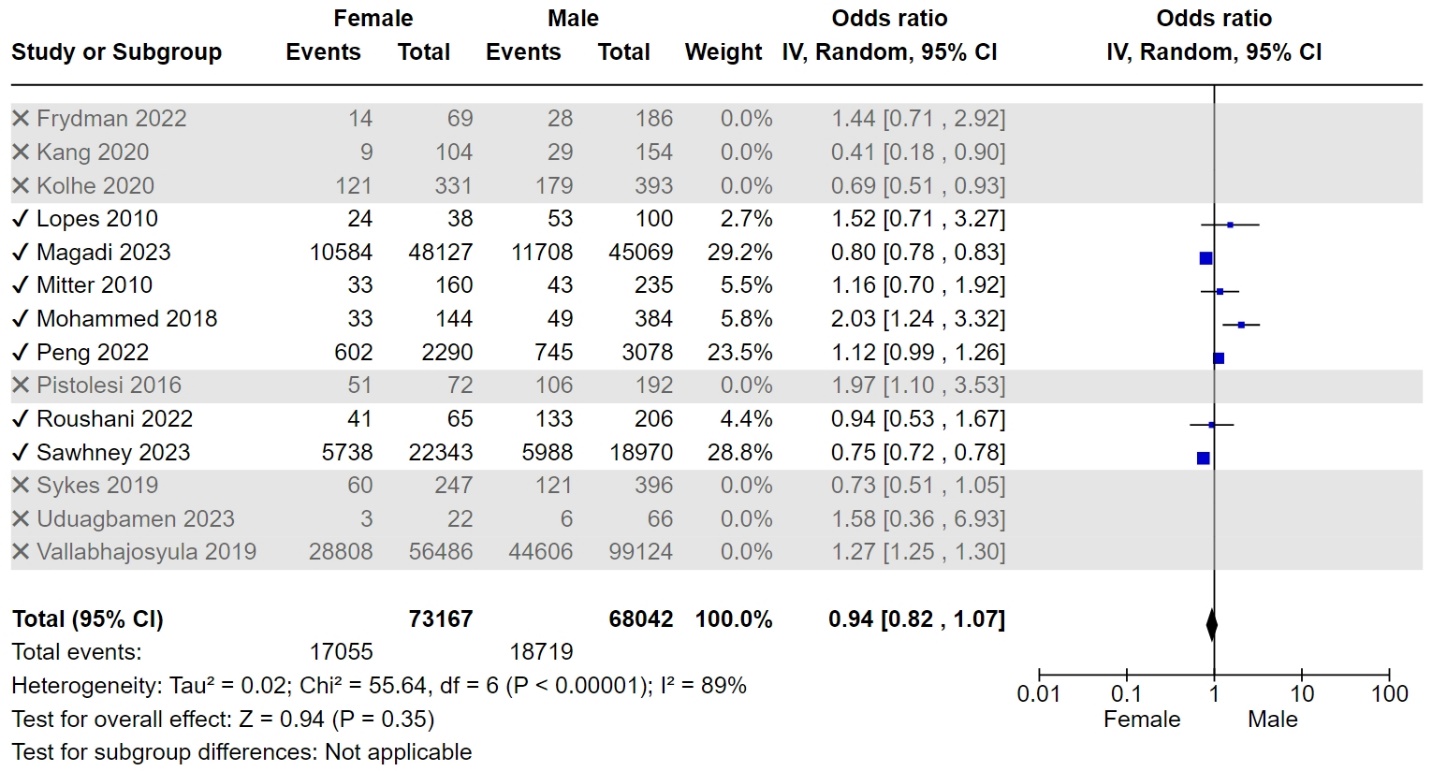


**Supplementary Figure S2: Forest plot of a random effects meta-analysis of all-cause mortality following at least one episode of AKI by sex / gender with subgroup analysis by risk of bias**


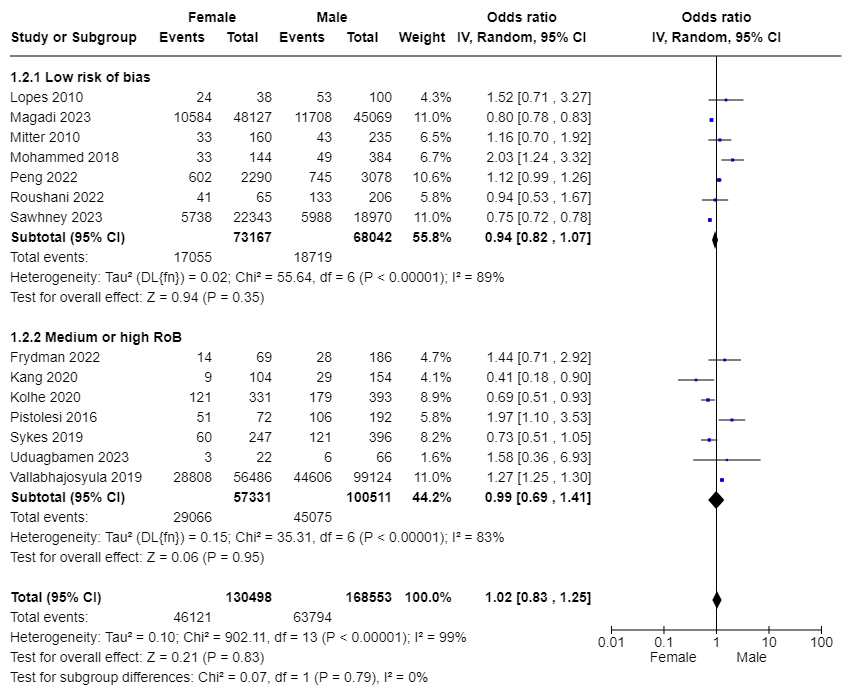


**Supplementary Figure S3: Funnel plot of log odd ratios for mortality following AKI by sex/gender***


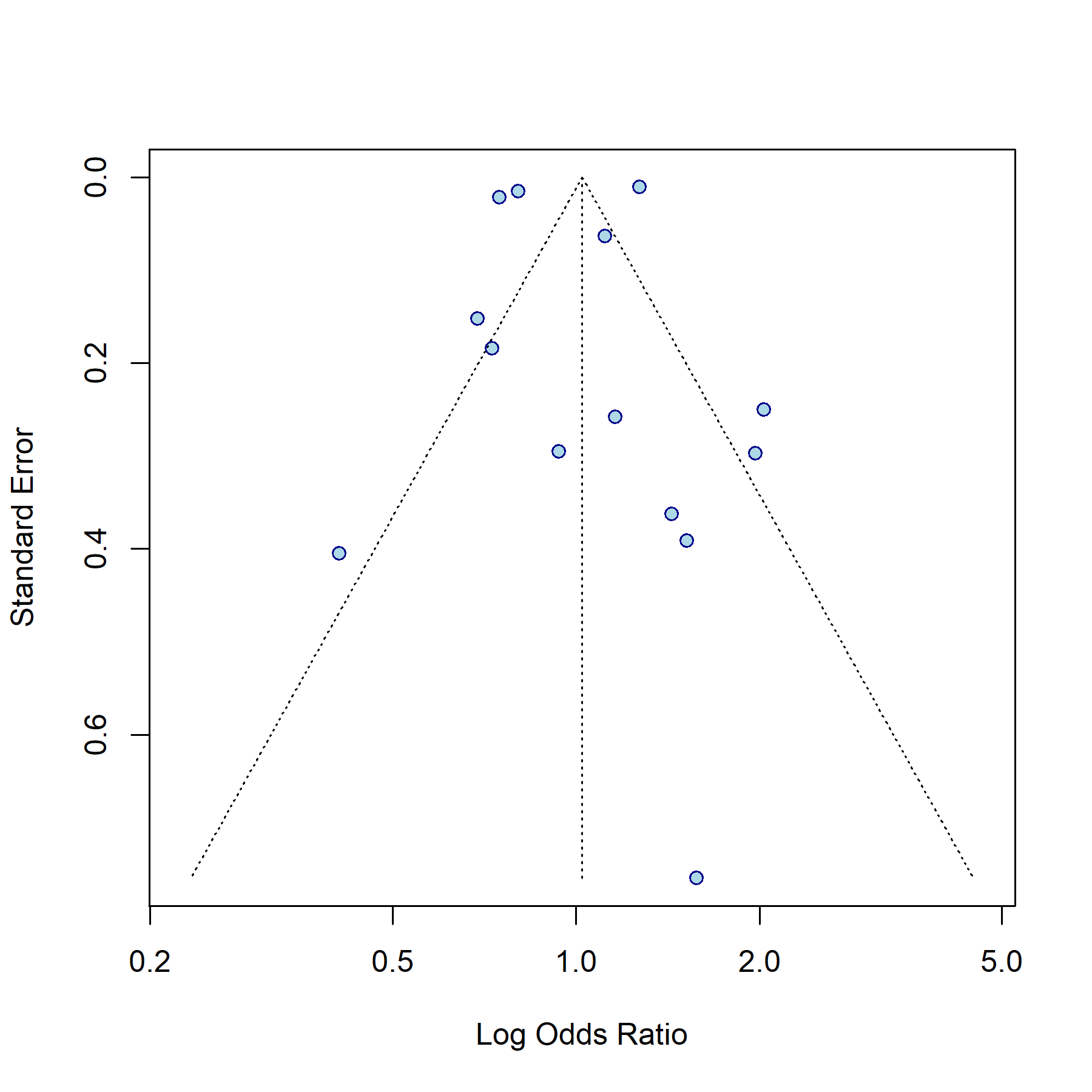

Supplement: Supplementary file 1 — Supplementary Material 1 [file 12882_2025_4391_MOESM1_ESM.docx]
